# Attenuation of Heparan Sulfate Proteoglycan Binding Enhances *In Vivo* Transduction of Human Primary Hepatocytes with AAV2

**DOI:** 10.1016/j.omtm.2020.05.004

**Published:** 2020-05-13

**Authors:** Marti Cabanes-Creus, Adrian Westhaus, Renina Gale Navarro, Grober Baltazar, Erhua Zhu, Anais K. Amaya, Sophia H.Y. Liao, Suzanne Scott, Erwan Sallard, Kimberley L. Dilworth, Arkadiusz Rybicki, Matthieu Drouyer, Claus V. Hallwirth, Antonette Bennett, Giorgia Santilli, Adrian J. Thrasher, Mavis Agbandje-McKenna, Ian E. Alexander, Leszek Lisowski

**Affiliations:** 1 Translational Vectorology Research Unit, Children’s Medical Research Institute, The University of Sydney, Westmead, NSW 2145, Australia; 2 Great Ormond Street Institute of Child Health, University College London, London WC1N 1EH, UK; 3 Gene Therapy Research Unit, Children’s Medical Research Institute & The Children’s Hospital at Westmead, University of Sydney, Westmead, NSW 2145, Australia; 4 Commonwealth Scientific and Industrial Research Organisation (CSIRO), North Ryde, NSW 2113, Australia; 5 Department of Biochemistry and Molecular Biology, Center for Structural Biology, University of Florida, Gainesville, FL 32610, USA; 6 Discipline of Child and Adolescent Health, The University of Sydney, Sydney, NSW 2006, Australia; 7 Vector and Genome Engineering Facility, Children’s Medical Research Institute, The University of Sydney, Westmead, NSW 2145, Australia; 8 Military Institute of Hygiene and Epidemiology, Biological Threats Identification and Countermeasure Center, 24-100 Puławy, Poland

## Abstract

Use of the prototypical adeno-associated virus type 2 (AAV2) capsid delivered unexpectedly modest efficacy in an early liver-targeted gene therapy trial for hemophilia B. This result is consistent with subsequent data generated in chimeric mouse-human livers showing that the AAV2 capsid transduces primary human hepatocytes *in vivo* with low efficiency. In contrast, novel variants generated by directed evolution in the same model, such as AAV-NP59, transduce primary human hepatocytes with high efficiency. While these empirical data have immense translational implications, the mechanisms underpinning this enhanced AAV capsid transduction performance in primary human hepatocytes are yet to be fully elucidated. Remarkably, AAV-NP59 differs from the prototypical AAV2 capsid by only 11 aa and can serve as a tool to study the correlation between capsid sequence/structure and vector function. Using two orthogonal vectorological approaches, we have determined that just 2 of the 11 changes present in AAV-NP59 (T503A and N596D) account for the enhanced transduction performance of this capsid variant in primary human hepatocytes *in vivo*, an effect that we have associated with attenuation of heparan sulfate proteoglycan (HSPG) binding affinity. In support of this hypothesis, we have identified, using directed evolution, two additional single amino acid substitution AAV2 variants, N496D and N582S, which are highly functional *in vivo*. Both substitution mutations reduce AAV2’s affinity for HSPG. Finally, we have modulated the ability of AAV8, a highly murine-hepatotropic serotype, to interact with HSPG. The results support our hypothesis that enhanced HSPG binding can negatively affect the *in vivo* function of otherwise strongly hepatotropic variants and that modulation of the interaction with HSPG is critical to ensure maximum efficiency *in vivo*. The insights gained through this study can have powerful implications for studies into AAV biology and capsid development for preclinical and clinical applications targeting liver and other organs.

## Graphical Abstract

**Figure:**
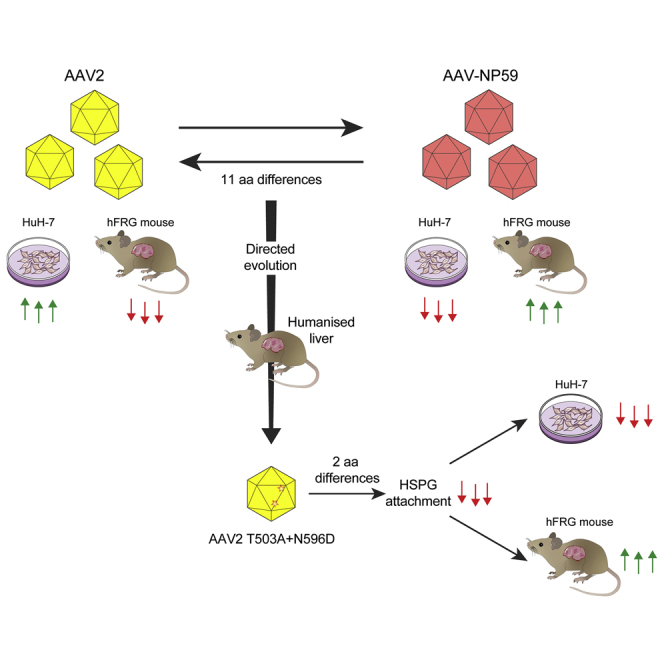

## Teaser

Adeno-associated viral vectors based on serotype 2 transduce primary human hepatocytes in a xenograft model of human liver with low efficiency. Lisowski and colleagues demonstrated that attenuation of the interaction of AAV2 with its primary receptor, heparan sulfate proteoglycan, significantly enhances transduction of human hepatocytes in this preclinical model.

## Introduction

The non-pathogenic adeno-associated virus type 2 (AAV2) is considered endemic in the human population, with serological evidence supporting lifetime infection rates of 30%–70% worldwide.^1^ Prototypical AAV2 was isolated in 1966 by Hoggan et al.^2^ as a contaminant of an adenovirus type 12 (strain 97838). Nearly 17 years passed before Srivastava et al.^3^ described the genome organization and the full nucleotide sequence of AAV2. Its single-stranded nature, size, and the presence of the 145-nt inverted terminal repeats (ITRs) were also determined. The genome of AAV2 contains two genes, the 5′ (*rep* open reading frame [ORF]) encodes four non-structural proteins (Rep78, Rep68, Rep52, and Rep40), important for genome replication and packaging into the virus capsid and acting as a transcriptional activator and repressor. The 3′ part (*cap* ORF) of the genome encodes three overlapping capsid viral proteins (referred to as VP1, VP2, and VP3).^3^ The AAV capsid is assembled from 60 copies of VP1/VP2/VP3 in a reported 1:1:10 ratio, respectively.^4^^,^^5^ In addition to the two main *rep* and *cap* ORFs, three additional nested ORFs within the *cap* gene have subsequently been discovered; one encodes the assembly-activating protein (AAP), the second encodes the X gene, and third encodes the membrane-associated accessory protein (MAAP).^6^, ^7^, ^8^ AAP is required for transport of the VPs from the cytoplasm to the nucleus where capsid assembly occurs. The MAAP was recently discovered and thought to play a role in the natural life-cycle of the virus, as well as the X gene product.^6^^,^^7^

Of the three AAV variants first discovered (AAV1–AAV3), AAV2 was the first variant to be successfully cloned into a bacterial plasmid (pSM620). This process allowed AAV production from HEK293-31 cells transfected with pSM620 and infected with human adenovirus 5.^9^ Soon afterward, AAV2 was used for the first time to deliver a DNA payload into mammalian cells (the process referred to as transduction).^10^ Flotte et al.^11^ pioneered the *in vivo* use of AAV2, successfully transducing rabbit lung tissue with an AAV vector encoding the cystic fibrosis transmembrane conductance regulator. The long-standing observation that the ITRs were the only elements required in *cis* for AAV replication and packaging facilitated the generation of the universal cross-packaging system composed of three plasmids, one containing the AAV *cap* gene of choice downstream of the AAV2 *rep* gene, and the other containing the transgene cassette cloned between the ITRs from AAV2, both complemented with a plasmid harboring the essential adenoviral genes to support AAV replication.^12^ This allowed researchers to quickly and conveniently package the same transgene cassettes into a variety of AAV capsids and enabled studies that demonstrated variant-specific transduction of target cells. Since AAV2 was the first variant vectorized, subsequently leading to the development of the AAV vector system based on the same variant, most studies related to the biology of this virus system have been carried out using prototypical AAV2.^13^ This includes identification of the first AAV cellular receptor, the membrane-associated heparan sulfate proteoglycan (HSPG), which was shown to mediate cellular attachment of AAV2,^14^ a finding that was later extended to other variants, such as AAV3, AAV6, and AAV13.^15^ Post-attachment interactions with the target cell remained less clearly defined until the recent identification of two highly conserved AAV entry receptors, AAVR^16^ and GPR108.^17^ The early interest in AAV2 as a potential gene therapy vector fueled studies that led to the identification of the residues involving HSPG binding. These studies showed that binding to HSPG involved direct interaction with arginine residues at positions 585 and 588, with contributions from R484, R487, and K532, the mutations of which decreased, but did not inhibit, binding to HSPG.^18^^,^^19^ Despite the importance of this canonical receptor for AAV2 biology, several groups have described HSPG-independent AAV2 attachment and internalization.^20^ Nevertheless, various levels of dependence on HSPG have been observed among cell types, and HSPG attachment has been reported to be essential for AAV2 transduction of some targets, such as primary murine hepatocytes.^21^

To date, the structures of AAV1-9 and rhesus isolates AAVrh8, AAVrh.10, AAVrh32.33 and AAVrh.39 have been determined by X-ray crystallography and/or Cryo-EM.^22^, ^23^, ^24^, ^25^, ^26^, ^27^, ^28^, ^29^, ^30^, ^31^, ^32^, ^33^, ^34^ The AAV capsid is a T = 1 icosahedron composed of 60 copies (in total) of overlapping VP1, VP2, and VP3, but only the structure of the common VP3 region is observed. This is likely due to the low copy number of VP1 and VP2 (~10% each) along with the predicted intrinsic disorder of the VP1/2 common region.^35^ The structure of the VP3 common region consists of an eight-stranded beta-barrel motif (βBIDG and βCHEF) and a conserved α helix (αA) that forms the interior surface and core of the virus capsid. Large loops interconnect the β strands and αA to form the exterior surface of the capsid. These loops differ in length and interact to generate the characteristic 2-fold depression, 3-fold protrusions, and a channel at the 5-fold axis of the capsid. Comparative analysis of the capsid structure and sequence of two diverse serotypes, AAV2 and AAV4, defined variable regions (VRs) within these loops.^24^ The VRs are responsible for functional variations between different AAV serotypes. These VRs dictate differential receptor binding phenotypes among the AAVs. Specifically, AAV2, AAV3, AAV6, and AAV13 use residues located in VR-V, VR-VI, and VR-VIII to bind HSPG.^30^^,^^36^^,^^37^.

Successful AAV2-based clinical trials in the eye^38^ resulted in the recent US Food and Drug Administration (FDA) approval of AAV2-based treatment for inherited retinal dystrophy (Luxturna [voretigene neparvovec], Spark Therapeutics). Intriguingly, the first liver-directed clinical trial, however, showed an unexpectedly poor efficacy of AAV2.^39^ This was even more surprising in light of the recent demonstration of an intimate evolutionary relationship between AAV2 and the human liver, as evidenced by detectable ongoing infection with AAV2-like viruses in ~10% of the studied human population,^40^ and the presence of binding sites for three human master hepatic transcription factors in the 3′ UTR of AAV2.^41^ Since the initial use of AAV2 in clinical studies targeting human liver,^39^ three additional natural isolates, AAV5, AAV8, and AAVrh.10, have been tested for liver-directed gene transfer, together with AAV-LK03, which is now being tested in phase III studies for hemophilia A.^42^ AAV-LK03 was selected through directed evolution of a diverse capsid library in the clinically predictive *Fah*^−/−^/*Rag2*^−/−^/*Il2rg*^−/−^ (FRG)^43^ mouse model, which is repopulated with primary human hepatocytes (humanized FRG [hFRG]).^44^ A recent similar selection method using the same mouse model has yielded two novel bioengineered capsids, AAV-NP40 and AAV-NP59, both of which can transduce primary human hepatocytes *in vivo* with high efficiency in the xenograft model of human liver.^45^ Remarkably, AAV-NP59 differs in only 11 aa from the prototypical AAV2, providing a unique opportunity to study the relationship between capsid sequence/structure and vector function. This could lead in turn to a better understanding of the structural determinants of efficient functional transduction of human hepatocytes.

In this study, we used AAV-NP59 as a reverse genetic tool to identify capsid residues that enhance transduction of human primary hepatocytes *in vivo*. Using two orthogonal vectorological approaches, we identified T503A and N596D substitutions to be the main determinants improving *in vivo* human hepatotropism of AAV-NP59 when compared to the prototypical AAV2. Counterintuitively, we show that these mutations reduce affinity of AAV-NP59 to HSPG, the primary receptor of AAV2, indicating that, contrary to what is observed for murine hepatocytes, HSPG attachment might not be a requirement for human hepatocyte transduction *in vivo*. In support of this hypothesis, we identified, using directed evolution, two additional AAV2 variants, N496D and N582S, which were highly functional *in vivo*. Both point mutations reduced affinity to HSPG. Finally, we modulated the ability of AAV8, a highly murine-hepatotropic serotype, to interact with HSPG. The results support our hypothesis that enhanced HSPG binding can negatively affect the *in vivo* function of otherwise strongly hepatotropic variants and that modulation of the interaction with HSPG is critical to ensure maximum efficiency *in vivo*. The insights gained through this study can have powerful implications for studies into AAV biology and capsid development for preclinical and clinical applications targeting liver and other organs.

## Results

### Functional Differences between AAV-NP59 and AAV2 Are Attributable to All, or a Subset of, 11 aa Substitutions

A recently identified bioengineered AAV variant, AAV-NP59, functionally transduces primary human hepatocytes in a xenograft model of human liver with significantly higher efficiency than that for AAV2.^45^ Interestingly, sequence analysis at the DNA level revealed that NP59 differed from AAV2 at 51 positions, 37 of which were silent (Figures S1A and S1B). The remaining 14 changes resulted in 11 aa differences between NP59 and prototypical AAV2 (Table S1). Thus, AAV-NP59 is a novel variant that can serve as a tool to study the correlation between capsid sequence/structure and vector function.

**Figure 1:**
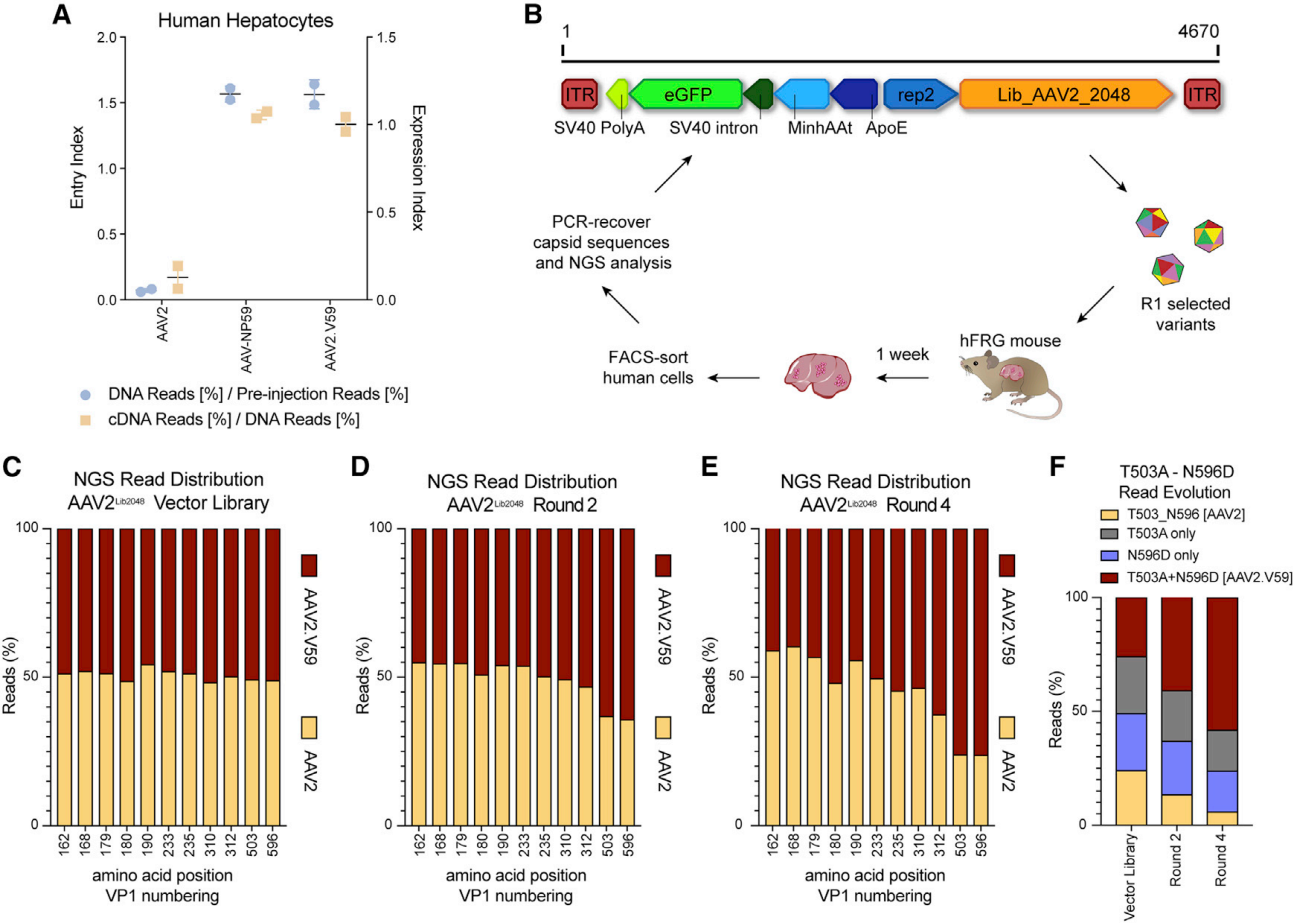
Validation and *in vivo* selection of Functional Transduction AAV2^Lib2048^ library. (A) *In vivo* comparison of physical and functional transduction of AAV2, AAV-NP59, and AAV2.V59 capsids in the xenograft liver model. Each AAV variant was used to package two unique barcoded ssAAV-LSP1-GFP-BCWPRE-BGHpA cassettes, and an equimolar mix of all three variants was used. NGS reads mapped to each capsid in human hepatocytes at the DNA level (cell entry, physical transduction, 113.6 vg/diploid human genome) normalized to the pre-injection are shown (entry index). cDNA reads (expression, functional transduction) normalized to the mapped DNA reads are also shown (expression index). (B) Functional transduction (FT) selection platform and the FT selection scheme. The capsid libraries are cloned downstream of the 3′ *rep* region with the *cap* expression driven by the p40 promoter. The LSP1-EGFP reporter cassette is positioned in the reverse direction to the p40 *cap*. (C–E) NGS analyses of amino acid distribution at the 11 positions variable between AAV2.V59 and AAV2, in the packaged library (C), after round 2 (D), and after round 4 (E) of the selection process. (F) NGS read distribution of AAV2 amino acid positions 503 and 596 on the initial packaged library and two subsequent rounds of selection.

To ensure that the observed *in vivo* functional differences between AAV-NP59 and prototypical AAV2 were attributable to all, or a subset, of the 11 aa changes and not to the genotypic context of the variable nucleotide positions, we generated an AAV2 variant with the 11 aa from AAV-NP59 (referred to as AAV2.V59). AAV2, AAV-NP59, and AAV2.V59 were used to package two independent barcoded (BC) reporter constructs expressing EGFP under the control of a liver-specific promoter (LSP)^46^ (ssAAV-LSP1-GFP-BC-WPRE-BGHpA). No significant differences in vector packaging efficiency were observed between AAV2.V59 and AAV-NP59, and both variants produced significantly higher yields than did prototypical AAV2 (Figure S2). Importantly, functional analysis in a xenograft mouse model of human liver revealed that AAV2.V59 transduced primary human hepatocytes with the same efficiency as AAV-NP59, both at the DNA (cell entry, physical transduction) and RNA (transgene expression, functional transduction) levels (Figure 1A). To enable a more robust analysis of data generated, we defined two indexes, the entry index (EI) and the expression index (EXI). EI corresponds to the quotient of capsid-specific next-generation sequencing (NGS) reads at the DNA level and the mapped NGS reads in the original vector mix, and it defines the relative ability of a given capsid to physically transduce the targeted cells. EXI corresponds to the barcode-specific ratio of cDNA mapped reads to DNA mapped reads and offers a relative view of the functional transduction of each capsid variant. These data indicated that the 11 aa were crucial for the significant functional difference between AAV2 and AAV-NP59 in the context of primary human hepatocytes. We next identified which of the 11 aa were necessary and sufficient to enhance AAV2’s liver-directed functional transduction by taking two orthogonal approaches: permutational and cluster analysis.

### Permutational Analysis Indicates That Functional Differences between AAV2 and AAV-NP59 Are Driven by 2 aa

We generated a binary capsid library (AAV2^Lib2048^) containing all possible permutations (n = 2^11^ = 2048) of AAV2- and AAV2.V59-specific residues at the 11 variable positions. To prevent formation of additional random changes in the *cap* gene due to replication-driven *in vivo* evolution, the AAV2^Lib2048^ library was cloned into a replication-incompetent functional transduction selection platform encoding an LSP1-EGFP reporter cassette (Figure 1B). This library platform design allows selection based on transgene expression (functional transduction) and thus to identify variants that, similar to AAV-NP59, could functionally transduce human hepatocytes with high efficiency. Analysis of the starting library using Illumina NGS confirmed the intended binary composition at each position (Figure 1C), while full-length *cap* sequencing of n = 27 randomly selected clones confirmed the binomial distribution of the AAV2^Lib2048^ library (Figure S3). The function transduction (FT)-AAV2^Lib2048^ library underwent four rounds of iterative *in vivo* selection on primary human hepatocytes in humanized xenograft FRG mice,^43^ as schematically depicted in Figure 1B. Illumina sequencing of the capsid regions containing the 11 key positions after rounds 2 and 4 revealed a positive selection of AAV2.V59 residues at positions 503 (T503A) and 596 (N596D) (Figures 1D and 1E). The remaining positions showed a preference for residues from AAV2 (aa 162, 168, 190, and 235), or showed no strong preference (aa 179, 180, 233, and 310). The residue at 312 showed a mild preference in favor of AAV2.V59 (Figure 1E). Importantly, further analysis revealed that clones harboring the double mutation (T503A+N596D) showed the strongest fold enrichment, suggesting a synergistic functional effect between both amino acids (Figure 1F). These results point to T503A and N596D as the key amino acid changes driving the observed *in vivo* differences between AAV2 and AAV2.V59.

### Structure-Driven Analysis Confirms the Results of the Permutational Analysis

**Figure 2:**
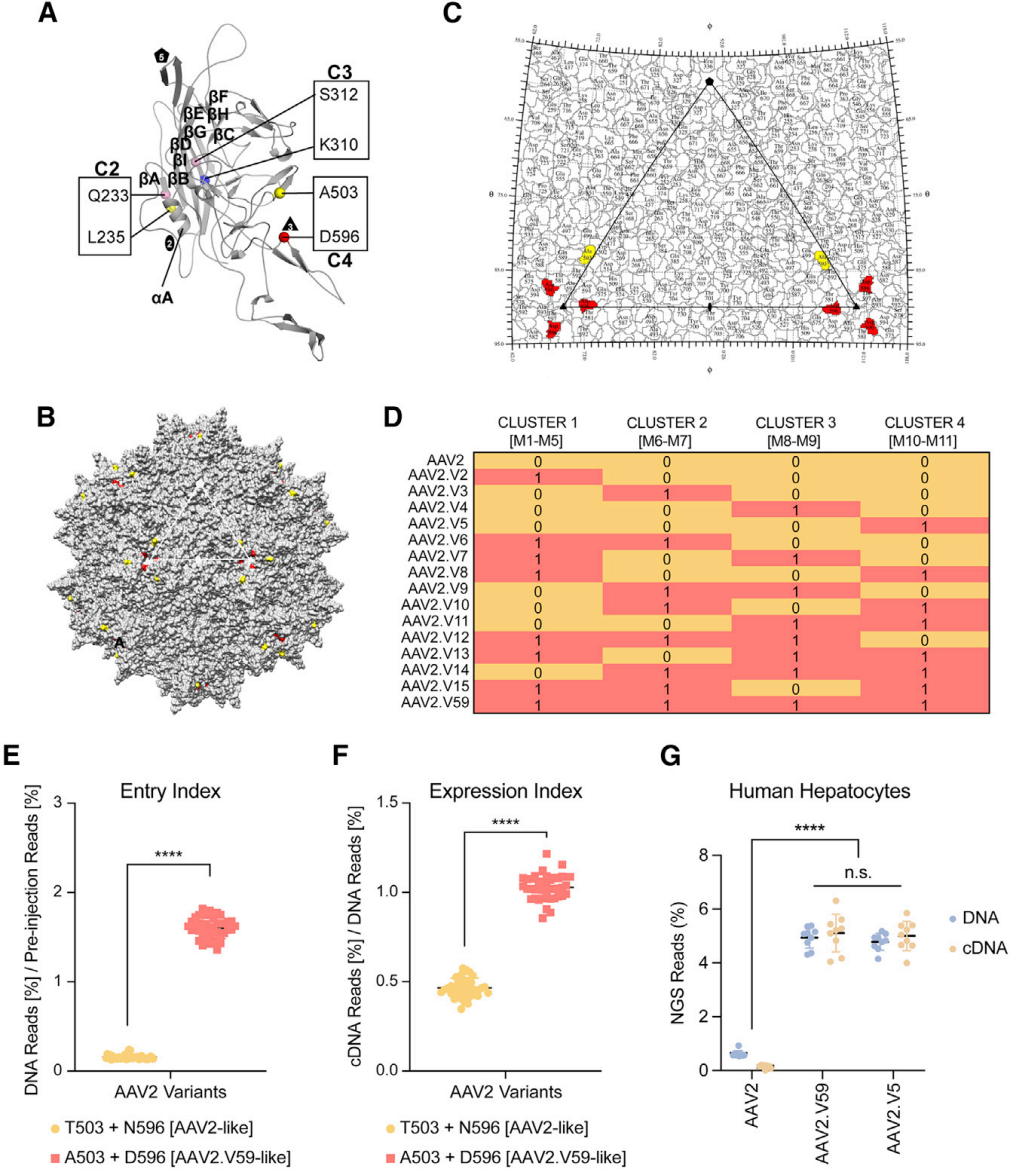
Functional test of AAV2.V59 variants in humanised FRG mice. (A) Structure and location of AAV2.V59 residues. Model of VP3 monomer colored gray. The residues in the VP3 clusters are shown as spheres, and the clusters are labeled as cluster 2 (C2), cluster 3 (C3), and cluster 4 (C4). (B and C) Surface map (B) and stereographic roadmap projection (C) of the 3D model viewed down the icosahedral 2-fold axis. The icosahedral 2-, 3-, and 5-fold axes, are depicted as an oval, a triangle, and a pentagon respectively. The non-polar residues L235 and A503 are colored yellow, polar residues Q233 and S312 are colored pink, basic residue K310 is colored blue, and acidic residue D596 is colored red. (D) Cluster composition of 16 AAV2 variants, including AAV2 and AAV2.V59. (See Figure S1 for composition of each cluster.) A yellow shadowed “0” indicates AAV2 origin for the whole cluster, whereas a shadowed salmon “1” indicates NP59 origin of the given cluster. (E and F) *In vivo* performance of the 16 AAV2 cluster variants in the humanized FRG (hFRG) model grouped by the origin of cluster 4 (AAV2 origin, yellow; NP59 origin, salmon). The results are shown as mean ± SD. (E) Entry index (2,014 vg/diploid human genome) and (F) the expression index. (G) *In vivo* comparison of AAV2, AAV2.V59, and AAV.V5 variants based on physical and functional transduction in the xenograft liver model. Percentage of NGS reads mapped to each capsid in human cells at the DNA (248 vg/diploid cell) and cDNA levels, normalized to the pre-injection mix, are shown. The results are shown as mean ± SD. Statistical significance was calculated using the two-tailed Mann-Whitney test. ∗∗∗∗p < 0.0001. n.s., not significant (p > 0.05).

In a parallel approach, structural *in silico* analysis of AAV2 and AAV-NP59 revealed that the 11 differing amino acid residues were localized in four distinct structural clusters (Figures 2A–2C; Figure S1B; Table S1). This provided an opportunity to investigate which structural clusters were responsible for the observed functional differences between AAV2 and AAV-NP59. To this end, we generated 16 AAV variants, where each clone harbored either the whole structural cluster from AAV2 or AAV2.V59, as depicted in Figure 2D. The AAV2 VP1 unique (VP1u) region (residues 1–137) does not contain any of the residues from the clusters, and the VP1/2 common region (residues 138–202) contains five residues (aa162, aa168, aa179, aa180, and aa190) that are a part of cluster 1. These two regions of the AAV VP are not observed in any available high-resolution structures of the AAVs. Clusters 2, 3, and 4 are a part of the VP3 common region (residues 203–735). The N terminus of VP3 (residues 203–215) has also not been observed in any available high-resolution AAV structure. Cluster 2 contains two residues (aa233 and aa235) located on αA of the VP3 (Figure 2A). This is located on the wall of the icosahedral 2-fold depression. Cluster 3 contains two residues (aa310 and aa312) located in β strand A, which forms part of the core of the capsid facing the capsid interior (Figure 2A). Cluster 4 contains two residues (aa503 and aa596) located in VR-V and VR-VIII, respectively. These VRs interact to form the 3-fold protrusion on the external surface of the capsid, and this region has been shown to be important for AAV antigenic reactivity, sialic acid binding, and HSPG binding (Figures 2A–2C).^47^^,^^48^

To eliminate animal-to-animal variability, we adapted an AAV *in vivo* biodistribution analysis method based on the NGS of vector-encoded DNA/RNA barcodes.^49^, ^50^, ^51^ Packaging of individually barcoded transgenes into multiple capsids enables a fast and powerful characterization of variants based on physical (cell entry, DNA) and functional (transgene expression, RNA/cDNA) transduction, respectively. To minimize the possibility that a particular 6-nt barcode affected transgene expression, five different barcodes were packaged per capsid (exemplified in Figure S4). Furthermore, to achieve a complete view of capsid functionality, the barcoded cassettes were packaged at increasing concentrations, allowing simultaneous study of vector performance at different multiplicities of transduction (MOTs) (Figure S4).

Each of the 16 AAV2 variants defined in Figure 2D was used to package five barcoded transgenes (see Materials and Methods for details). Study of the barcode distribution in the vector mix confirmed the presence of capsid transgene subpopulations at increasing concentrations (Table S2). The vector mix was subsequently injected into an hFRG mouse (1 × 10^11^ total vector genomes [vg]/animal), and the barcoded region composition was analyzed at the DNA and RNA/cDNA levels in human hepatocytes 1 week after injection. As expected from findings from the permutational analysis (Figure 1F), the entry and the expression indexes of the variants clustered according to the origin of the fourth structural cluster containing both T503A and N596D mutations (Figures 2E and 2F; Figure S5). Specifically, all vectors with high human liver tropism *in vivo* contained both T503A and N596D changes (from AAV2.V59), whereas all eight vectors exhibiting lower transduction of primary human hepatocytes contained AAV2 residues at those positions (Figures 2E and 2F). Interestingly, no difference on entry was observed on murine liver cells (Figure S5C). As shown in Figure S6, the rate of transcription (DNA to cDNA) appears to be linear and affected by the capsid used. Specifically, variants harboring the T503A and N596D mutations (AAV2.V5 and AAV2.V59) showed a significantly enhanced functional transduction compared to variants harboring AAV2 residues at the same positions.

The two independent approaches (permutational analysis and cluster analysis) identified the T503A and N596D mutations as the key determinants driving the improved performance of AAV2.V59 in primary human hepatocytes in the humanized liver model. As a final validation of these findings, we injected an hFRG mouse with AAV2, AAV2.V59, and AAV2.V5 (AAV2 T503A+N596D), with each encoding n = 9 barcoded transgenes at increasing concentration. As shown in Figure 2G, no statistically significant difference was observed between AAV2.V59 and the AAV2.V5 at the DNA or cDNA level. The percentage of reads for any of these two variants including T503A and N596D substitutions was significantly higher than AAV2, in human primary hepatocytes. Based on these results, AAV2.V5, which differed from prototypical AAV2 at only those two positions, was used in subsequent mechanistic studies, in which we determined how the 2 aa residues affect the capsid performance and unlock the ability of AAV2 to functionally transduce primary human hepatocytes with high efficiency.

### Amino Acid Changes at Capsid Positions T503A and N596D Reduce Heparin Binding

**Figure 3:**
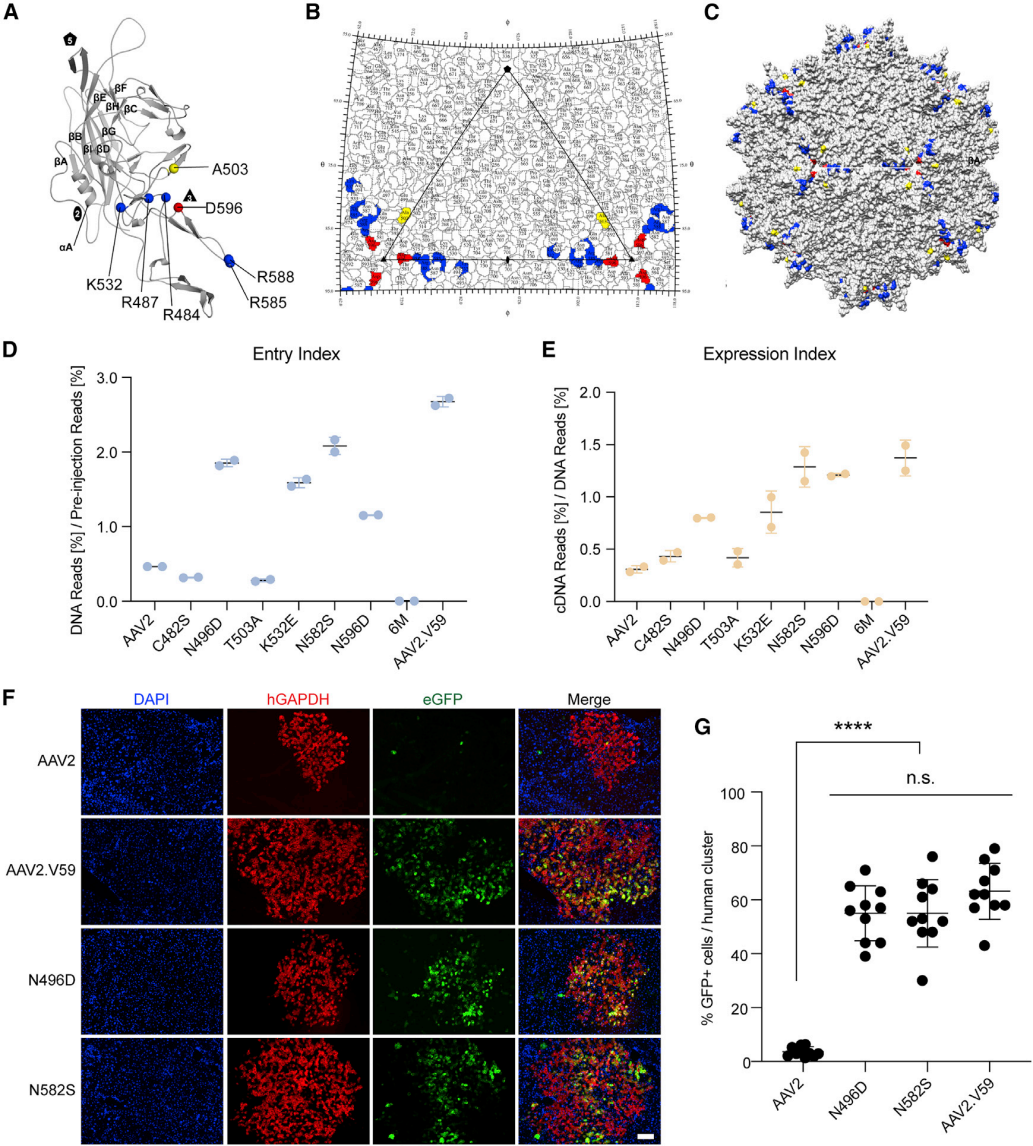
Fnctional analysis of HSPG-detargeted AAV2 variants in the hFRG mice. (A–C) Structure and location of AAV2.V5 residues. (A) Model of VP3 monomer colored gray. The residues involved in heparin binding are R484, R487, K532, R585, and R588, and they are colored blue. Two residues important for reduced heparin binding and improved hepatotropic transduction are D596 (red) and A503 (yellow). (B and C) Surface map (B) and stereographic roadmap projection (C) of the 3D model viewed down the icosahedral 2-fold axes. The icosahedral 2-fold, 3-fold, and 5-fold axes are depicted as an oval, triangle, and pentagon, respectively. (D and E) Relative *in vivo* performance of AAV2 variants in the hFRG model, represented as (D) entry (499.3 vg/diploid cell) and expression (E) indexes. (F) Representative immunohistochemical analysis of the liver of an hFRG mouse transduced with AAV2, AAV2.V59, AAV2-N5496D, and AAV2-N582S variants. Statistical significance was calculated using the two-tailed Mann-Whitney test, comparing the performance of each novel variant with AAV2. Red, human GAPDH; green, vector-encoded GFP; blue, DAPI (nuclei). Scale bar, 100 μm. (G) Quantification of the percentage of transduced human hepatocytes per human cluster. Data are shown as mean ± SD (n = 10 human clusters/mouse, n = 1 mouse/vector). Statistical significance was calculated using the two-tailed Mann-Whitney test. ∗∗∗∗p < 0.0001. n.s., not significant (p > 0.05).

Strong binding to HSPG by AAV2 lowers transduction and decreases the spread of this serotype in the brain.^52^^,^^53^ Interestingly, similar to the HSPG-binding domain (HBD) of AAV2, residues 503 and 596 of AAV2.V5 are located within the 3-fold capsid protrusions adjacent to the determinant residues (Figures 3A–3C). The T503A substitution, which removes a polar side chain, is part of the VR-V surface loop and is located on the wall of the 3-fold protrusion facing the 2-/5-fold wall and should not, in theory, affect HSPG binding (Figures 2B and 3B). However, T503 along with structurally adjacent E499 and K507 form part of the footprint for the recently discovered trafficking AAV receptor (AAVR).^54^ In addition, structurally equivalent residue T502 in AAV1 along with adjacent W503 play a role in sialic acid binding for this serotype,^55^ and this capsid pocket is also involved in galactose binding for AAV9.^56^ N596D, which is also not a canonical HSPG-binding residue, introduces a net negative charge adjacent to R484 and is located on the capsid surface at the base of the protrusions surrounding the 3-fold axis and adjacent to HSPG-binding residues (Figures 3A–3C). To investigate whether these changes affected HSPG binding, the binding affinities of AAV2, AAV2.V5, AAV2.V12, and AAV2.V59 (Figure 2D) were compared using a HiTrap heparin column. AAV2 and AAV2.V12 eluted at a similar and higher salt concentration than did AAV2.V59 and AAV2.V5 (Table 1; Figures S7–S10), suggesting that T503A+N596D changes present in AAV2.V5 and AAV2.V59 are responsible for reduced interaction with heparin. Importantly, when the set of 16 variants containing all possible combinations of the structural clusters (Figure 2D) was tested on the human hepatocellular carcinoma cell line HuH-7, two distinct groups of vectors could be identified based on their ability to functionally transduce the cells (Figure S11). Interestingly, and in contrast to data obtained on primary human hepatocytes in the hFRG mouse (Figures 2E and 2F), the vectors containing cluster 4 residues, and thus residues at position 503 and 596 from AAV2, were more efficient at transducing HuH-7 cells than those capsids that contained cluster 4 residues from AAV-NP59 (Figure S11). An *in vitro* competition assay using soluble heparin confirmed that AAV2.V59 and AAV2.V5 were more resistant to free heparin than were AAV2-derived vectors, whereas AAV2 and AAV2.V12 showed similar inhibition profiles (Figure S12). Notably, no noticeable differences in binding to AAVR^16^ were observed between AAV2, AAV2.V5, and AAV2.V59 despite the T503A change (Figure S13). Combined, the data strongly suggest that the weaker binding of AAV2.V59 to HSPG, as compared to AAV2, is responsible for its improved function in primary human hepatocytes *in vivo* in the FRG model. Interestingly, vector yield analysis revealed that all the variants carrying A503+D596 residues showed significantly higher yields during production than vectors containing AAV2 residues at these positions (Figure S14).

**Table 1:** Summary of HiTrap Heparin Column Binding Studies

AAV Capsid	AAV Predominantly Detected in:	[NaCl] at Elution Peak Maxima (mM)
AAV2	elution fraction	453
AAV2.V5 (AAV2 T503A+N596D)	elution fraction	371
AAV2.V12 (AAV2.V59 A503T+D596N)	elution fraction	463
AAV2.V59	elution fraction	368
AAV2-N496D	elution fraction	200
AAV2-N582S	elution fraction	362
AAV2-K532E	elution fraction	282
AAV2-6M	flowthrough	–
AAV8	flowthrough	–
AAV8-RQNR	elution fraction	650
AAV8-E533K	elution fraction	369
AAV8-RQNR-N499D	elution fraction	460
AAV8-E533K-N499D	elution fraction	271

### Alternative Substitutions Attenuating Heparin Binding Improve *In Vivo* Functional Transduction of Primary Human Hepatocytes with AAV2

Given the high performance of the bioengineered vectors selected from the shuffled AAV library used by Paulk et al.,^45^ we investigated whether other functional capsid variants present in that same library could provide additional insights into the relationship between capsid sequence and function on primary human hepatocytes. To do so, the same shuffled capsid library used by Paulk et al. was cloned into our functional transduction selection platform (Figure 1B), and the library was selected on primary human hepatocytes in the FRG xenograft liver model following the previously described protocol (Figure 1B). NGS analysis of AAV2 amino acid positions 474–617 after three rounds of selection led to the identification of six residues that underwent enrichment (C482S, N496D, T503A, K532E, N582S, N596D; Figure S15A). Two of the six residues were already described for AAV-NP59 (T503A, N596D), while a third one (K532E) is present in another human hepatotropic variant, AAV-NP40.^45^ From the substitutions that underwent enrichment, three (N496D, K532E, and N582S) corresponded to AAV2 positions described to directly interact with HSPG,^36^^,^^47^ two (T503A and N596D) do not interact directly with HSPG but were shown to collectively affect heparin binding (Table 1; Figures S6–S9), while one (C482), located at the 3-fold axis, is not on the capsid surface. To study the effect of the six mutations on vector function, AAV2 variants carrying each of the individual point mutations were tested. In addition, a seventh variant that combined all six individual changes, referred to as AAV2.6M, was also tested. Functional comparison of these seven variants in the hFRG xenograft model, using AAV2 and AAV2.V59 as negative and positive controls, respectively, revealed that three of the substitutions, N496D, K532E, and N582S, were sufficient to substantially enhance both the entry and expression indexes of AAV2 in human hepatocytes (Figures 3D and 3E). Inclusion of all six changes in a single variant (AAV2.6M) had a detrimental effect on the *in vivo* performance in both murine and human hepatocytes, and T503A hampered murine cell entry (Figures 3D and 3E; Figure S15B). The *in vivo* performance of AAV2-N496D and AAV2-N582S were further confirmed using immunohistochemistry (Figures 3F and 3G). Investigation of whether the observed functional outcomes were related to HSPG binding showed that the N496D and N582S (as well as K532E) variants had decreased affinity for heparin (Table 1; Figures S16–S18), supporting the HSPG binding modulation hypothesis. Alternatively, AAV2.6M was found solely in the flowthrough, which could explain its weak performance in the functional assay (Figures 3D and 3E; Figure S19).

### Functional Transduction by AAV8 Can Be Controlled via Modulation of Heparin Affinity

**Figure 4:**
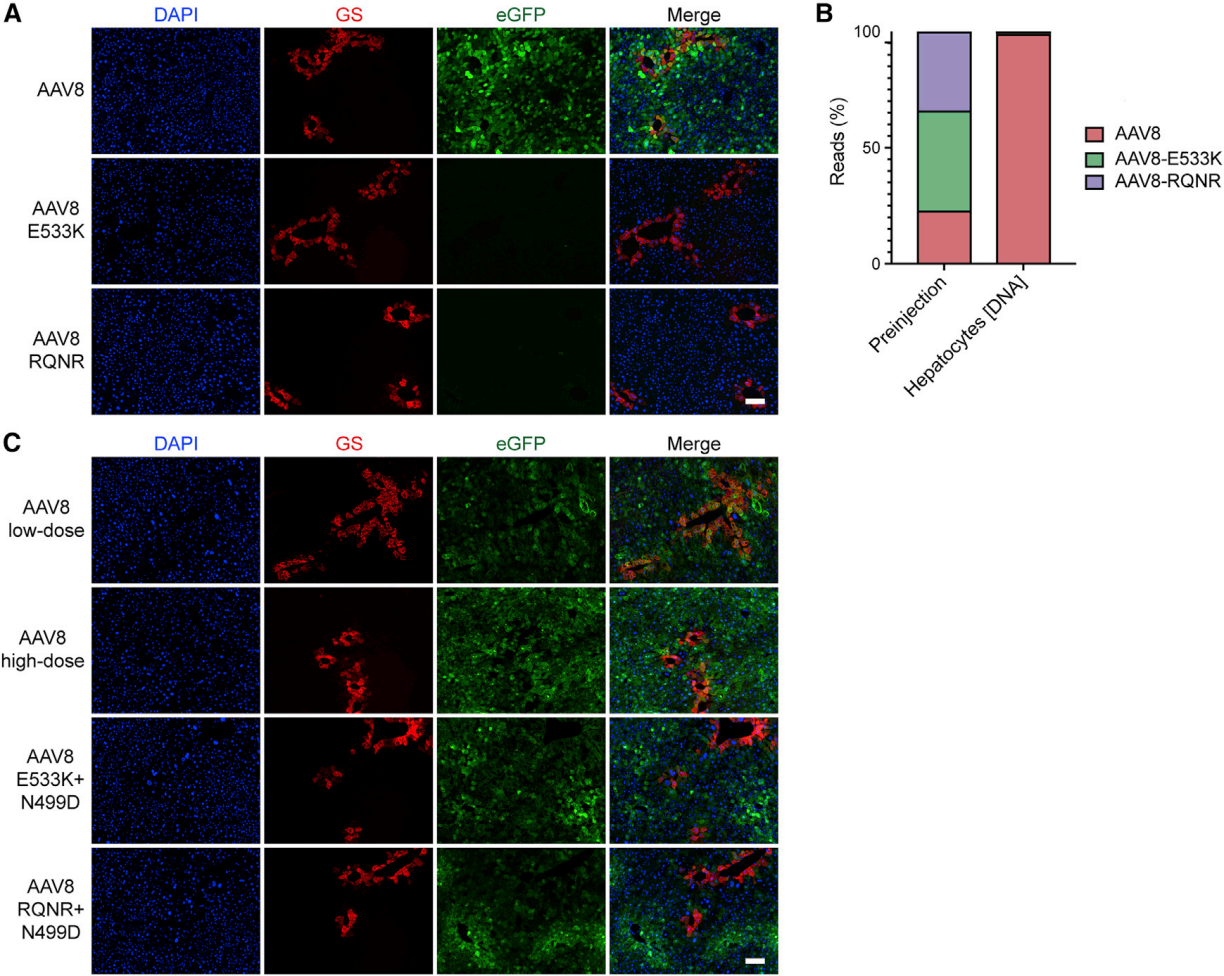
In vivo functional test of AAV8 variants on non-engrafted FRG mice. (A) Representative immunohistochemical analysis of a naive FRG mouse liver transduced with AAV8, AAV8-E533K, and AAV8-RQNR variants encoding the ssAAV-LSP1-GFP-WPRE-BGHpA construct (1 × 10^10^ vg/mouse). Red, glutamine synthetase; green, vector-encoded GFP; blue, DAPI (nuclei). Scale bar, 100 μm. (B) NGS read distribution of AAV8 variants expressed as a percentage of total mapped reads, in the pre-injection AAV mix and in the DNA recovered from murine liver cells. (C) Representative immunohistochemical analysis of the liver from a naive FRG mouse transduced with AAV8, AAV8-E533K-N499D, and AAV8-RQNR-N499D variants encoding a scAAV-CAG-GFP-SV40pA construct. To enable comparison of the AAV8 transduction pattern, AAV8 was injected at 5 × 10^10^ vg/mouse (high dose) and 5 × 10^9^ vg/mouse (low dose). Red, glutamine synthetase; green, vector-encoded GFP; blue, DAPI (nuclei). Scale bar, 100 μm.

We next investigated whether the observed HBD alterations and associated functional effects were restricted to AAV2 or could be expanded to other serotypes. To do so, we generated two AAV8 mutants, AAV8-E533K and AAV8-RQNR, previously shown to have increased affinity for heparin,^57^^,^^58^ and assessed them using a HiTrap heparin column. As expected, AAV8 was found in the flowthrough (Figure S20) while the two variants eluted at NaCl concentrations similar to (AAV8-E533K) or higher than AAV2 (AAV8-RQNR) (Table 1; Figures S21 and S22). Following this validation on the heparin column, the AAV8 variants encoding the previously used ssAAV-LSP-GFP-BC-WPRE-BGHpA cassette were functionally tested in naive male FRG mice. In contrast to AAV8, AAV8-E533K and AAV8-RQNR showed no detectable murine hepatocyte transduction at the dose tested (1 × 10^10^ vg/mouse) (Figure 4A). To confirm the results and account for any potential mouse-to-mouse variations, the three variants were used to package two barcoded LSP-GFP-BC cassettes per capsid and were co-injected into a naive FRG mouse, together with AAV8 as a positive control. NGS analysis of AAV genomes recovered from murine hepatocytes confirmed that both AAV8 variants with increased heparin-binding capacity (AAV8-E533K and AAV8-RQNR) were strongly de-targeted from this organ (Figure 4B). Having shown that AAV8-E533K and AAV8-RQNR lost the ability to transduce murine hepatocytes compared to parental AAV8, we investigated whether a single amino acid substitution could reduce the heparin affinity of AAV8-E533K and AAV8-RQNR and rescue their performance *in vivo*, as observed for AAV2.V5 and AAV2. To do so, we introduced an N-to-D substitution at position 499, *in silico* predicted as the structural equivalent of AAV2-N496D, onto the heparin-binding AAV8 variants (referred as to AAV8-E533K-N499D and AAV8-RQNR-N499D). A heparin-binding assay confirmed the anticipated reduction in heparin affinity (Table 1; Figures S23 and S24). As shown in Figure 4C, the N499D mutation improved the ability of the AAV8 HSPG mutants to transduce murine hepatocytes, although, in contrast to AAV8, these variants appeared to transduce periportal hepatocytes with higher efficiency.

## Discussion

Improved human hepatotropism of AAV vectors is required to bring a higher proportion of liver diseases that are theoretically amenable to gene therapy within the technological reach of AAV-mediated gene transfer. The considerable recent advances in the development of functionally superior capsid variants, however, have not been paralleled by equal progress in elucidating the mechanisms that underpin these advances. In this study, we used a sequence comparison between the AAV2 capsid and the bioengineered AAV-NP59 variant to understand the capsid-based determinants of *in vivo* human hepatotropism. These studies linked the superior *in vivo* performance of AAV-NP59 with reduced binding affinity to heparin, the experimental surrogate for HSPG. We show that in the context of AAV-NP59 this effect is driven solely by two amino acid substitutions, T503A and N596D. AAV2-T503A has been previously shown to improve *in vitro* transduction when compared to AAV2,^59^ while AAV2-N596D was reported as a heparin-de-targeted variant.^60^ Nevertheless, the synergestic effect of the combined amino acid substitutions on human liver transduction has gone unrecognized.

In fact, the deliberate attenuation of HSPG binding has been reported by two independent groups to increase AAV transduction of the murine central nervous system (CNS) and the retina.^52^^,^^53^ In the first study, the key AAV2 arginine residues at positions 585 and 588 were mutated to alanine (R585A, R588A), generating the AAV2-HBKO variant. This bioengineered variant displayed significantly greater photoreceptor transduction following subretinal delivery and widespread striatal and cortical expression following intrastriatal delivery in mice than did the parental AAV2. Importantly, AAV2-HBKO was found to be de-targeted from the murine liver, and a substantial improvement in gene transfer to the heart and skeletal muscle was also observed.^52^ AAV2-HBKO was also recently reported to outperform prototypical AAV2 at the levels of both transduction and intratissue spreading in non-human primate CNS.^61^ The second study described a bioengineered AAV2-like variant, AAV-TT, incorporating amino acid residues conserved among AAV2-like variants isolated from primary human samples, most notably R585S and R588T mutations, which abolished HSPG binding.^53^ This variant also exhibited strong tropism for the CNS, as well as minimal transduction of other organs, such as the liver, when tested in rodents.^53^ Similar observations were reported in 2006 by Büning and colleagues^21^ who generated a library of AAV2 capsids carrying insertions of seven randomized amino acids at position 587 and stratified them according to their affinity for heparin. Non-binding AAVs were de-targeted from the murine spleen and liver, with concomitantly elevated levels of viral DNA in the blood. The authors interpreted this to imply non-specific, HSPG-mediated retention of HSPG-binding AAVs in the liver and spleen, which could be linked to the high levels of HSPG expression both on the cell surface and on the extracellular matrices of these organs.^62^ Similarly, two recent AAV2-related studies from the Muzyczka group^63^ and the Church group^7^ have also described decreases in murine liver transduction upon mutation of R585 and R588 residues. Importantly, all of these studies were limited to the use of murine models and were, therefore, not configured to provide insights into improved human hepatotropism, while, in accordance with our data, human hepatocyte entry appears to be HSPG-independent. Thus, based on our results, we propose a functional model, in which upon reduction of the affinity of AAV2 toward HSPG, (1) vector sequestration on extracellular matrixes is reduced, (2) the concentration of free vector increases, and (3) the biodistribution of the vector increases, leading to (4) increased transduction of human hepatocytes in an HSPG-independent process. The final parameter of the model, which requires further investigation and validation, relates to the higher normalized expression (expression index) observed for the HSPG de-targeted variants compared to HSPG-binding counterparts (Figure 2E). Our data strongly suggest that, besides biodistribution and cellular entry, strong HSPG binding through direct or indirect mechanisms, such as those affecting intracellular trafficking, negatively affects post-entry steps leading to transgene expression, further lowering the overall functional efficiency of HSPG-binding vectors. Interestingly, we did not detect any effect on murine liver transduction as shown in Figure S5. All variants, regardless of cluster 4 origin, appeared to physically transduce cells at equal efficiencies. This could be related to the fact that T503A and N596D reduce, but not eliminate, HSPG binding.

A valuable insight for the preclinical testing and selection of capsid candidates intended for use in the human liver is the dichotomous performance of AAV2-like and NP59-like variants *in vitro* and *in vivo* (compare Figures 2E and 2F and Figure S11). This further underscores the importance of preclinical testing in biologically predictive model systems, since the *in vitro* results using human hepatocyte-derived cell lines would be misleading in terms of clinical performance. It is logical to hypothesize that due to the lack of HSPG-rich extracellular matrix affecting capsid biodistribution, increased HSPG binding is beneficial for the *in vitro* performance of AAV2. An interesting question that arises is why the prototypical AAV2 would present such a high affinity to HSPG if this property was theoretically detrimental for *in vivo* spreading. Others have hypothesized that the ability of AAV2 to bind HSPG could constitute a tissue culture adaptation acquired during serial passaging in the presence of adenovirus *in vitro*.^64^ Importantly, this artificial property could also directly contribute to the low yields in the purification process typically observed for AAV2, as it has been previously proposed that strong binding to HSPG could lead to the loss of vector particles in the cell debris.^65^ The fact that all the AAV2 variants with decreased HSPG binding yielded a higher number of packaged particles per cell than did AAV2 (Figure S14) supports this hypothesis. The data further suggest that vectors with decreased HSPG binding will enhance translational studies through improved function and enhanced manufacturing, potentially facilitating therapeutic benefits at a lower vector dose (improved safety) and lower cost per patient (improved healthcare impact). However, given the high sequence identity between these variants and AAV2, and the high prevalence of anti-AAV2 neutralizing antibodies in the human population,^1^ further developments to decrease the antibody recognition might be required to maximize clinical impact.^66^^,^^67^

Interestingly, the second most functional AAV variant described by Paulk et al.,^45^ AAV-NP40, which, similarly to AAV-NP59 is also closely related to AAV2 (12 aa differ between the variants), does not harbor the key substitutions (T503A and N596D) responsible for improved hepatotropism of AAV-NP59. Guided by our increased understanding of the relationship between HSPG binding and vector hepatotropism, we investigated whether AAV-NP40 harbored any substantial changes within the HBD situated at the capsid 3-fold protrusions. Pairwise alignment of this variant with AAV2 revealed that AAV-NP40 carries a lysine for glutamate change on K532E (AAV2 VP1 numbering), one of the five key amino acids that has historically been associated with HS interaction. The third variant, also published by Paulk et al.,^45^ AAV-NP84, carries an additional potentially HSPG de-targeting substitution at the key arginine 585 (R585G). Thus, the data suggest that the three variants share a common mechanism underlying their improved functional performance on primary human hepatocytes. Importantly, selection of the same capsid library used by Paulk et al. using our replication-incompetent selection platform yielded enrichment of variants harboring T503A, K532E, and N596D mutations, suggesting that these variants likely existed in the initial capsid pool within the library. However, the R585G substitution present in AAV-NP84 likely arose from a *de novo* mutation acquired during library replication, since enrichment of this mutation was not observed in our study, implying that this variant was not present in the initial library. Our selection approach identified three additional enriched substitutions (C482S, N496D, and N582S), two of which were shown to substantially improve human hepatotropism of AAV2 (Figures 3D–3G). Since the *in vivo* human hepatocyte cell entry of the AAV2-C482S variant was found to be similar to that of prototypical AAV2 (Figure 3D), we hypothesize that the enrichment of this variant was likely a consequence of a process unrelated to HSPG de-targeting. The AAV capsid proteins have five conserved cysteine residues, with C482 being the most variable (C482S/C482M changes in AAV4, AAV5, and AAV9).^68^ Previous studies have shown that the performance of the AAV2-C482S variant was indistinguishable from AAV2 in terms of capsid formation, titer, and transduction efficiency.^68^ Thus, the enrichment of this variant was possibly due to a synergistic effect linked to another enriched residue, such as T503A+N596D.

Notably, the same phenotypical properties that apply to AAV2 variants could be translated to another AAV variant, as shown for AAV8. The generation of the two AAV8 heparin-binding variants (AAV8-E533K and AAV8-RQNR) resulted in (1) lower vector yield per packaging cell (Figure S25) and (2) reduced murine hepatotropism, presumably due to hampered biodistribution. Reduced functional transduction of murine hepatocytes was previously reported for an AAV8 variant with an added heparin-binding domain, as well as reduced yields for an AAV9 variant with a similar heparin-binding related modification.^69^ Importantly, through the introduction of the structurally equivalent N496D mutation onto the AAV8 heparin-binding variants, we were able to correlate the reduction of heparin binding with the partial recovery of murine hepatotropism (Figure 4C). Interestingly, and inversely to prototypical AAV8, which has been shown to preferentially transduce pericentral hepatocytes in the murine liver,^70^^,^^71^ the zonation profile of AAV8-RQNR-N499D and AAV8-E533K-N499D variants appeared to be periportal (Figure 4C). We hypothesize that this change in transduction zonation is related to the fact that these two variants still bind HSPG, in juxtaposition to the non-binding AAV8 (Table 1). Under our working model, HSPG binding would artificially favor transduction of hepatocytes more proximal to the blood vessels through which AAVs enter the liver, similarly to what was reported for AAV2.^70^ This observation has important consequences for selecting a specific AAV variant for gene therapy applications that would benefit from targeting of specific hepatic zones, such as for metabolic defects of urea cycle disorders (UCDs).

Finally, while the data presented in this study are of direct relevance to the development of new AAV-mediated treatment options for diseases of the human liver, the targeted attenuation or modulation of AAV capsid interactions with HSPG may be similarly applicable to other organs, especially when applied to parental serotypes in addition to AAV2. Various tissues all present a unique HSPG environment, and, therefore, fine-tuning the strength of AAV capsid interactions with HSPG could prove an essential step in optimizing the functional performance of capsids intended for therapeutic use.

## Materials and Methods

### Mouse Studies

All animal experimental procedures and care were approved by the joint Children’s Medical Research Institute (CMRI) and The Children’s Hospital at Westmead Animal Care and Ethics Committee. *Fah*^−/−^*Rag2*^−/−^*Il2rg*^−/−^ (FRG) mice^43^ were bred at CMRI. Recipient animals were housed in individually ventilated cages (IVCs) with 2-(2-nitro-4-trifluoromethylbenzoyl)-1,3-cyclohexanedione (NTBC)-supplemented drinking water. When 6–8 weeks old, FRG mice were engrafted with human hepatocytes (Lonza Group, Basel, Switzerland), as described previously.^43^ hFRG mice were placed on 10% NTBC 1 week prior to AAV transduction and were maintained in this condition until harvest. Information on sex, age, and levels of repopulation of the mice used in this study can be found in Table S5. Mice were randomly assigned to experiments and transduced via intravenous injection (lateral tail vein) with the indicated vector doses. Mice were euthanized by CO_2_ inhalation either 1 or 2 weeks after transduction for barcoded NGS analysis or immunohistochemistry studies, respectively.

### Isolation of Human Hepatocytes by Collagenase Perfusion

To obtain murine and human single-cell suspensions from xenografted murine livers, the inferior vena cava (IVC) was cannulated and the portal vein cut to allow outflow of perfusate. 25 mL of four solutions was pumped with an osmotic minipump (Gilson Minipuls 3) in the following order: 25 mL of Hanks’ balanced salt solution (HBSS) (−/−) (catalog no. H9394; Sigma), 25 mL of HBSS/0.5 mM EDTA, 25 mL of HBSS, and 25 mL of HBSS/5 mM CaCl_2_, 0.05% (w/v) collagenase IV (Sigma), and 0.01% (w/v) DNase I (Sigma). Following perfusion, liver capsules were broken with the blunt end of a scalpel blade on a Petri dish containing 25 mL of DMEM supplemented with 10% fetal bovine serum (FBS). The suspension was passed through a 100-μm nylon cell strainer and spun at 50 × *g* for 3 min at 4°C. Live and dead cells were separated with isotonic Percoll (GE Healthcare), following the manufacturer’s instructions. Live cells were pelleted at 860 × *g* for 10 min at 4°C and resuspended in FACS buffer (PBS (−/−) with 5% FBS and 5 mM EDTA). Cells were labeled with biotin-conjugated anti-mouse-H2Kb (clone AF6-88.5, BD Pharmingen, 553568; 1:100) and allophycocyanin (APC)-conjugated streptavidin (eBioscience, 17-4317-82; 1:500) for murine labeling and with phycoerythrin (PE)-conjugated anti-human-HLA (human leukocyte antigen)-ABC (clone W6/32; Invitrogen, 12-9983-42; 1:20) for human hepatocyte labeling. GFP-positive labeled samples were sorted to a minimal 95% purity using a BD Influx cell sorter. Sorting of the GFP-positive population was included to enrich for murine hepatocytes among non-parenchymal cells (NPCs), given the hepatocyte-restricted expression of the pLSP1-GFP-WPRE-BGHpA AAV construct. Flow cytometry was performed at the Flow Cytometry Facility, Westmead Institute for Medical Research (Westmead, NSW, Australia). The data were analyzed using FlowJo 7.6.1.

### Human Albumin ELISA

Levels of human cell engraftment in engrafted FRG mice were measured assessing the presence of human albumin on peripheral blood, using the human albumin ELISA quantitation kit (Bethyl Laboratories, catalog no. E80-129).^43^

### Adeno-Associated Virus Transgene Constructs

All of the vectors used in the study contain AAV2 ITR sequences. The AAV construct pLSP1-EGFP-WPRE-BGHpA, which encodes EGFP under the transcriptional control of a heterologous promoter containing one copy of the *SERPINA1* (hAAT) promoter and two copies of the *APOE* enhancer element, has been previously reported.^72^ 6-nt-long barcodes were cloned downstream of EGFP.

### DNA and RNA Isolation

For DNA extraction, sorted cells were resuspended in 200 μL of lysis buffer (100 mM Tris-HCl [pH 8.5]; Astral Scientific, BioSD8141-450ML), 5 mM EDTA (Thermo Fisher Scientific), 0.2% (w/v) sodium dodecyl sulfate (Sigma-Aldrich), and 200 mM NaCl (Sigma-Aldrich) containing 50 μg/mL proteinase K (Bioline) and incubated overnight at 56°C, followed by addition of PureLink RNase A (Thermo Fisher Scientific, catalog no. 12091021) at 0.4 μg/μL and incubation at 37°C. DNA was then extracted using a standard phenol/chloroform protocol using phenol/chloroform/isoamyl alcohol (25:24:1) (Sigma-Aldrich), followed by DNA ethanol precipitation.^73^ RNA was extracted using the Direct-zol kit (Zymogen, catalog no. R2062) and subsequently treated with TURBO DNase (Thermo Fisher Scientific, catalog no. AM2238). cDNA was synthesized using the SuperScript IV first-strand synthesis system, following the manufacturer’s instructions (Thermo Fisher Scientific, catalog no. 18091050).

### AAV Vector Packaging and Viral Production

AAV constructs were packaged into AAV capsids using HEK293 cells and a helper-virus-free system as previously described.^74^ Genomes were packaged in capsid variants using packaging plasmid constructs harboring *rep* genes from AAV2 and a specific *cap.* Packaging of multiple barcoded ss-LSP1-EGFP-BC-WPRE-BGHpA transgenes at increasing concentration was achieved by simultaneous transfection of 2, 4, 8, 12, and 16 μg of single-barcoded transgenes per vector production (5 × 15-cm HEK293T plates). Functional transduction libraries were packaged as described above, with the additive co-transfection of 37.5 μg/vector production of a plasmid harboring *rep2* (p-Rep2). All vectors/libraries were purified using iodixanol gradient ultracentrifugation as previously described.^75^ AAV preparations were titered using real-time quantitative PCR (qPCR) using *EGFP*-specific qPCR primers GFP-qPCR-For/Rev (Table S4).

### Cell Culture, Vector Transduction, and Heparin Competition Assay

HuH-7 cells were kindly provided by Dr. Jerome Laurence (University of Sydney). HEK293 cells were obtained from ATCC. All cells were tested for mycoplasma and were mycoplasma-free. Cells were cultured in Dulbecco’s modified Eagle’s medium (DMEM) (Gibco, 11965-092) supplemented with 100 U/mL penicillin/100 μg/mL streptomycin (Sigma-Aldrich, P4458) and 10% FBS (Sigma-Aldrich, F9423-500mL, lot no. 16K598). For HuH-7, media were also supplemented with non-essential amino acids (Gibco, 11140-050). Cells were passaged using TrypLE express enzyme (Gibco, 12604-21). For transduction studies, cells were plated into 24-well plates in complete DMEM at 1 × 10^5^ cells per well and incubated overnight in a tissue-culture incubator at 37°C/5% CO_2_. 16 h later, the vector stock was added to cells (at the indicated vg copies [vgc]/cell). For the heparin competition assay (Figure S12), cells were seeded at 10^5^ per well into 24-well plates at day 0 and transduced at the indicated vgc/cell. When indicated, heparin sodium salt (Sigma, H3149-50KU, lot no. SLBW2119) was supplemented from a 100× stock at 100 μg/mL (Figures S12A and S12B) or at 400 μg/mL (Figures S12C and S12D). After 72 h, the cells were harvested using TrypLE express and analyzed for GFP using BD LSRFortessa cell analyzer. The data were analyzed using FlowJo 7.6.1.

### Barcode Amplification, NGS, and Distribution Analysis

The 150-bp region englobing the 6-nt barcode was amplified with Q5 high-fidelity DNA polymerase (NEB, catalog no. M0491L) using BC_F and BC_R primers (Table S4). NGS library preparations and sequencing using a 2 × 150-paired-end (PE) configuration were performed by Genewiz (Suzhou, China) using an Illumina MiSeq instrument. To process reads and count barcodes we used a Snakemake (5.6) pipeline.^76^ Paired reads were merged using BBMerge and filtered for reads of the expected length in a second pass through BBDuk, both from BBTools 38.68 (https://sourceforge.net/projects/bbmap/). Merged, filtered fastq files were passed to a Perl (5.26)^77^ script that matched barcodes corresponding to AAV variants.

### Immunohistochemical Analysis of Mouse Livers

Engrafted and non-engrafted mouse livers were fixed with 4% (w/v) paraformaldehyde and cryo-protected in 10%–30% (w/v) sucrose before freezing in OCT (Tissue-Tek; Sakura Finetek USA, Torrance, CA, USA), as previously described.^72^ Livers were sectioned (5 μm) and permeabilized in −20°C methanol and then room temperature 0.1% Triton X-100. Sections were stained with DAPI (Invitrogen, D1306) at 0.08 ng/mL and anti-human GAPDH antibody (Abcam, catalog no. ab215227, clone AF674). When indicated, sections were also reacted with anti-glutamine synthetase antibody (Abcam, catalog no. ab73593). Following immunolabeling, the images were captured and analyzed on a Zeiss Axio Imager M1 using ZEN 2 software. Percentages of transduced human hepatocytes per field of view were determined by counting total human GAPDH-positive cells and EGFP/human GAPDH double-positive cells.

### Site-Directed Mutagenesis

Site-directed mutagenesis was performed using the Q5 site-directed mutagenesis kit (NEB, catalog no. E0554S). All the AAV *cap* variants generated via site-directed mutagenesis and the specific primers used for each are summarized in Table S3.

### Sanger Sequencing

Sanger sequencing was performed at the Garvan Molecular Genetics facility of the Garvan Institute of Medical Research (Darlinghurst, NSW, Australia).

### Heparin Binding Assay

The heparin affinity of listed AAV vector variants was determined on an ÄKTA pure 25 M2 (GE Healthcare) fast protein liquid chromatography (FPLC) system using a 1-mL HiTrap heparin HP column (GE Healthcare, catalog no. 1704601, lot no. 10276193). All chromatography steps were performed at a flow rate of 1 mL/min at room temperature. 7 × 10^11^ vg of iodixanol gradient-purified recombinant AAV (rAAV) vector encoding an LSP1-EGFP-BC-WPRE-BGHpA were diluted in a dilution buffer containing 2.5 mM KCl, 1 mM MgCl, and 10 mM phosphate (pH 7.4). This reduced NaCl concentration to a final 40 mM. Samples were then concentrated to a volume of 150 μL using an Amicon Ultra-15 centrifugal filter unit (Merck, catalog no. UFC910096) with a 10,000 kDa cutoff. The heparin column was routinely equilibrated with 3 column vol (CV) of buffer B (PBS + 1 M NaCl [pH 7.4]), followed by 5 CV of buffer A (40 mM NaCl, 2.5 mM KCl, 1 mM MgCl, 10 mM phosphate [pH 7.4]). rAAV was loaded into a 2-mL sample loop and 135 μL and applied to the heparin column with 4 CV of buffer A. The column was washed with 20 CV of buffer A to wash off unbound particles. For binding variants, the affinity of the serotype to heparin was determined by eluting the sample with a linear gradient of 0%–100% buffer B (40-1,137 mM NaCl), applied over 10 CV. The elution NaCl concentration was measured at the maximum of the UV absorbance (A280) peak for each rAAV variant. The flowthrough and elution phases were collected as 0.25-mL fractions using the Fraction Collection F9-C (GE Healthcare). The presence of rAAV in the A280 peaks was confirmed by running corresponding fractions on SDS-PAGE and silver staining to detect VP1, VP2, and VP3 proteins, and the overall proportion of rAAV molecules on the flowthrough/elution phases were determined by SYBR Green qPCR (Bio-Rad, catalog no. 172-5125) using GFP-qPCR-For and GFP-qPCR-Rev primers (Table S4).

### Vector DNA Copy Number Per Cell

Vector copy numbers were measured via digital droplet PCR (ddPCR, Bio-Rad, Berkeley, CA, USA) using EvaGreen supermix (Bio-Rad, catalog no. 1864034) and following the manufacturer’s instructions. To detect AAV genomes, GFP primers were used (GFP-qPCR-For/Rev), and vector genomes were normalized to human albumin copy number using primers human ALB_F/R_ddPCR (Table S4).

### AAV Structural Visualizations

To visualize the location of cluster residues on the AAV2.V59 and AAV2.V5 capsids, a 3D homology model of a VP3 monomer was generated by uploading the sequence to the online SWISS-MODEL server.^78^^,^^79^ A 60-mer of the VP3 was made using the oligomer generator subroutine of the online VIPERdb server.^80^ Visualization with the COOT application,^81^ Pymol,^82^ and Chimera^83^ showed that the location of the residues in clusters 2, 3, and 4. Stereographic roadmap projections were generated using the program RIVEM.^84^

### Data Sharing

The data that support the findings of this study are available from the corresponding author upon request.

### Statistical Analyses

Nonparametric statistical analyses were performed using the two-tailed Mann-Whitney test with the specified biological replicates in each experimental group. p < 0.05 was considered significant.

### Construction of Library AAV2^Lib2048^

For a detailed description of the process, refer to Figure S26. Four AAV2 backbone variants encoding for the full *cap* ORF were first generated (AAV2, a T503A variant, a N596D variant, and a double mutant). The DNA fragments englobing all of the possible combinations corresponding to the first five mutations between AAV2 and NP59 were custom synthesized (2^5^ = 32 fragments), as well as the following four (2^4^ = 16 fragments). Fragments were individually PCR amplified with overlapping primers and Gibson assembled on an equimolar ratio to the PCR-amplified and DpnI-treated backbones englobing the four distinct variants (2^2^ = 4). Thus, the total complexity of the library was expected to be of 2,048 variants (32 × 16 × 4), equivalent to the permutation of the 11 variable amino acids (2^11^ = 2,048). The assembled library was then electroporated into SS320 cells (Lucigen, catalog no. 60512-2). The pool of transformants was grown overnight in 250 mL of Luria-Bertani media supplemented with trimethoprim (Sigma-Aldrich, catalog no. T7883) (final concentration of 10 μg/mL). Total plasmids were purified with an EndoFree maxiprep kit (QIAGEN, catalog no. 12362) as per the manufacturer’s instructions and subsequently digested overnight with SwaI and NsiI. 1.4 μg of insert was ligated at 16°C using T4 DNA ligase (NEB, catalog no. M0202) for 16 h into 1 μg of the recipient functional transduction AAV2-based platform digested with compatible enzymes. Ligation reactions were concentrated using ethanol precipitation, electroporated into SS320 electrocompetent cells, and grown as described above. Library monitoring was achieved by individually amplifying capsid regions corresponding to clusters with primers cluster 1–4 (F/R, Table S4) followed by NGS (2 × 150 paired-ends for cluster 1–5, 2 × 350 paired-ends for cluster 4).

For library selection, 100 ng of purified DNA from sorted cells was then used for PCR recovery of the enriched full-length capsids using Q5 polymerase (NEB, catalog no. M0491S), Cap Rescue F/R primers (Table S4) and the following thermocycler conditions: 30 s at 98°C, 35 cycles of 10 s at 98°C, 60°C for 10 s, 72°C for 1.10 min, and a final extension of 72°C for 5 min. The PCR product was cloned into a compatible recipient plasmid upon Gibson assembly.

### Functional Transduction Library Selection

The original shuffled AAV library containing AAV-NP59 was kindly provided by Prof. Mark Kay (Stanford University). The library was digested overnight with SwaI and NsiI (flanking the *cap* gene) and cloned into the functional transduction plasmid platform as described above. Packaged functional transduction libraries were injected at 5 × 10^10^ vg/mouse. One week after transduction, GFP^+^ human hepatocytes were recovered and total DNA was extracted as described above. The *cap* gene was PCR recovered with primers Recovery_F/R (Table S4), and the amplicon was used to generate the subsequent library as described under “Construction of Library^Lib2048^” above.

### Construction of Clustered Variants and AAV2.V59

The fifteen clustered variants described in Figure 2B were built by individual Gibson based assemblies of the individually synthesized fragments defined for AAV2^Lib2048^, harboring either the whole cluster from AAV2 or from NP59 origin. As example, AAV2.V59 was generated assembling the three fragments harboring all the eleven mutations.

### Viral Overlay Assay

A virus overlay assay was performed as described before with minor modifications.^85^ HuH-7 membrane proteins were extracted using the Mem-PER Plus membrane protein extraction kit (Thermo Fisher Scientific, catalog no. 89842) as per the manufacturer’s instructions. 100 μg of purified membrane proteins was separated using 4%–12% NuPAGE Bis-Tris gels (Life Technologies, Carlsbad, CA, USA, catalog no. NP0322) and electrotransferred onto a polyvinylidene fluoride (PVDF) membrane. The membrane was sequentially incubated with TBST buffer (Tris-buffered saline with 0.1% Tween 20) with 5% non-fat milk (NFT) first and with purified rAAV vectors at 5 × 10^11^ vgc/mL in TBST-2% NFT overnight. Membrane was then washed three times (10 min/wash) with membrane wash buffer (1× PBS with 0.1% Tween 20) followed by incubation with an anti-intact AAV2 A20 antibody (ARP, 03-61055) at a 1:100 dilution for 1 h at room temperature in TBST-2% NFT. Membrane was then washed three times (10 min/wash) with membrane wash buffer. A horseradish peroxidase (HRP)-conjugated secondary antibody was used then to detect signal using SuperSignal West Pico chemiluminescent substrate (Thermo Fisher Scientific, Rockford, IL, USA, catalog no. 34080) and a FujiFilm luminescent image analyzer system (LAS-4000). Membrane was then stripped and incubated with anti-KIAA0319L (AAV-R) (Abcam, AB105385) at 1:400 dilution and signal was detected as described below.

## Author Contributions

M.C.-C., A.W., I.E.A. M.A.-M., and L.L designed the experiments. M.C.-C., A.W., R.G.N., G.B., E.Z., A.K.A., S.H.Y.L, S.S., E.S., K.L.D., A.R., M.D., C.V.H., A.B. and L.L. generated reagents, protocols, performed experiments, and analyzed data. M.C.-C., C.V.H., M.D. and L.L. wrote the article and generated the figures. All authors reviewed, edited, and commented on the article.

## Conflicts of Interest

M.C.-C., C.V.H., I.E.A., and L.L. are inventors on patent applications filed by Children’s Medical Research Institute related to AAV capsid sequences and *in vivo* function of novel AAV variants. L.L. is a co-founder and scientific advisor of LogicBio Therapeutics and the founding scientist of Perception Biosystems. A.J.T. is a co-founder and scientific consultant for Orchard Therapeutics, as well as a consultant for Rocket Pharmaceuticals, Generation Bio, bluebird bio, 4Bio Capital Partners, and Sana Biotechnology. M.A.-M. is a Scientific Advisory Board (SAB) member for Voyager Therapeutics, Inc., and AGTC, has a sponsored research agreement with StrideBio, Inc., Voyager Therapeutics, Inc., and Intima Biosciences, Inc., and is a consultant for Intima Biosciences, Inc. M.A.-M. is a co-founder of StrideBio, Inc., a biopharmaceutical company with interest in developing AAV vectors for gene delivery application. M.A.-M. and A.B. have intellectual property (IP) licensed to biopharmaceutical companies. L.L. and I.A.E. have consulted on technologies addressed in this paper. L.L. and I.A.E. have stock and/or equity in companies with technology broadly related to this paper. The remaining authors declare no competing interests.

## Supplemental Information

Document S1. Figures S1–S26, Tables S1–S5, and Supplemental Materials and Methods

Document S2. Article plus Supplemental Information
