# Supplementary material for: Attenuation of Heparan Sulfate Proteoglycan Binding Enhances In Vivo Transduction of Human Primary Hepatocytes with AAV2
Source: Mol Ther Methods Clin Dev. 2020 May 13;17:1139–54. doi: 10.1016/j.omtm.2020.05.004 (PMC7260615; doi:10.1016/j.omtm.2020.05.004)
Supplement: Document S1. Figures S1–S26, Tables S1–S5, and Supplemental Materials and Methods [file mmc1.pdf]

OMTM, Volume *17*

Supplemental Information

Attenuation of Heparan Sulfate Proteoglycan Binding Enhances *In Vivo* Transduction of Human Primary Hepatocytes with AAV2

Marti Cabanes-Creus, Adrian Westhaus, Renina Gale Navarro, Grober Baltazar, Erhua Zhu, Anais K. Amaya, Sophia H.Y. Liao, Suzanne Scott, Erwan Sallard, Kimberley L. Dilworth, Arkadiusz Rybicki, Matthieu Drouyer, Claus V. Hallwirth, Antonette Bennett, Giorgia Santilli, Adrian J. Thrasher, Mavis Agbandje-McKenna, Ian E. Alexander, and Leszek Lisowski

Supplementary Figure S1. DNA (A) and protein (B) sequence alignments of AAV2 and AAV-NP59. Structural clusters corresponding to residues described at Supplementary Table 1 are defined.

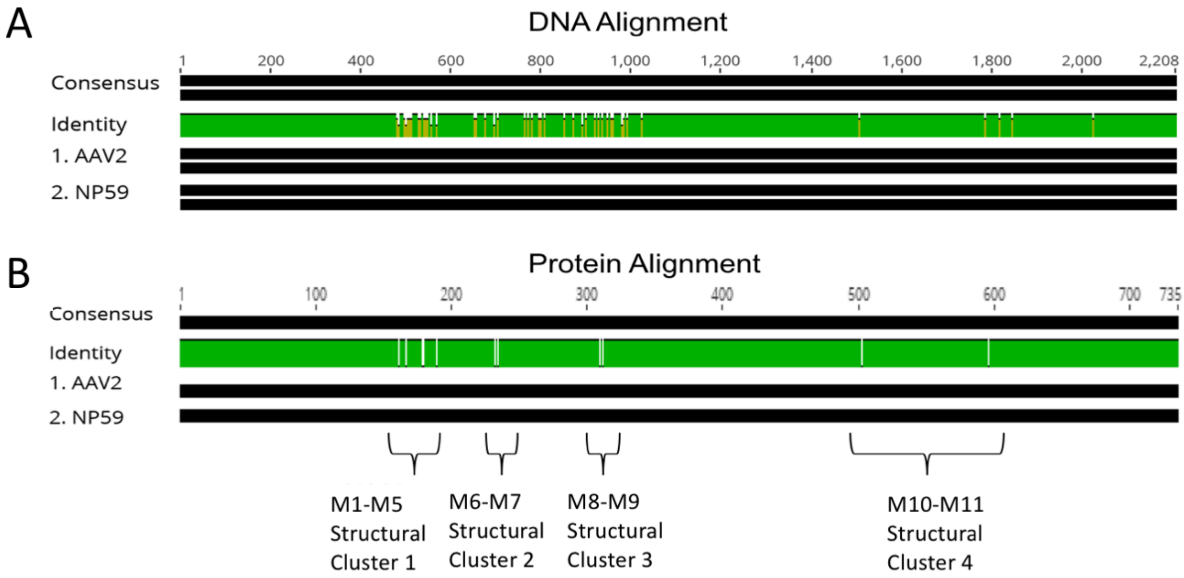

Supplementary Figure S2. AAV yield comparison from five independent crude productions using helper plasmids encoding for AAV2, AAV-NP59 and AAV2.V59.

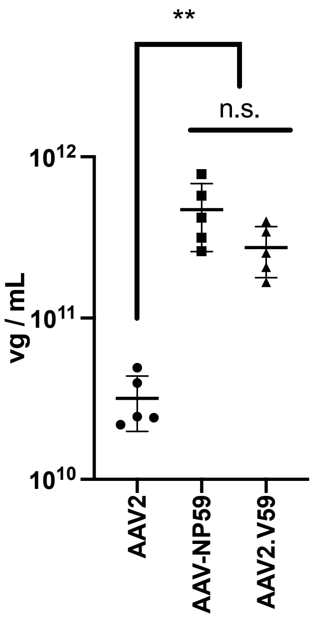

Supplementary Figure S3. Expected (orange) and observed (green) distributions of theoretical and fully sequenced (n=27) variants, respectively, of the binary capsid library AAV2^{Lib2048}. Accumulated mutations refer to total number of residue differences between the studied clone and AAV2. Out of the 2048 library variants, we expected only one to harbour 0 (AAV2) and one to harbour 11 (AAV2.V59).

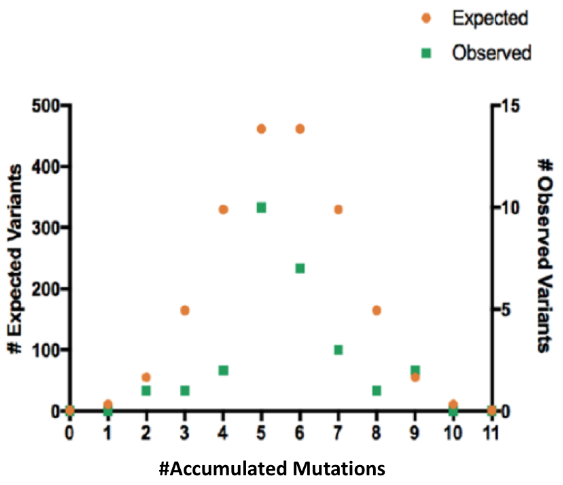

Supplementary Figure S4. Schematic representation of Entry and Expression Indexes exemplified with the study of two capsids. AAV capsid variants are individually packaged with five barcodes at increasing concentration (Capsid 1, BC1-5; Capsid 2, BC6-10). This results in a corresponding viral population at respective concentrations of barcodes. Both preparations are then individually tittered, mixed at 1:1 ratio (Viral mix) and co-injected into a single hFRG mouse. One week after injection, the chimeric liver is perfused and murine and human cells FACS sorted for DNA and RNA extraction. The barcoded region is then analysed on the initial viral mix, the DNA population and the RNA (cDNA) populations. For each barcode, the entry index corresponds to the quotient between the DNA reads and the viral mix, and the expression between the corresponding cDNA and DN mapped reads.

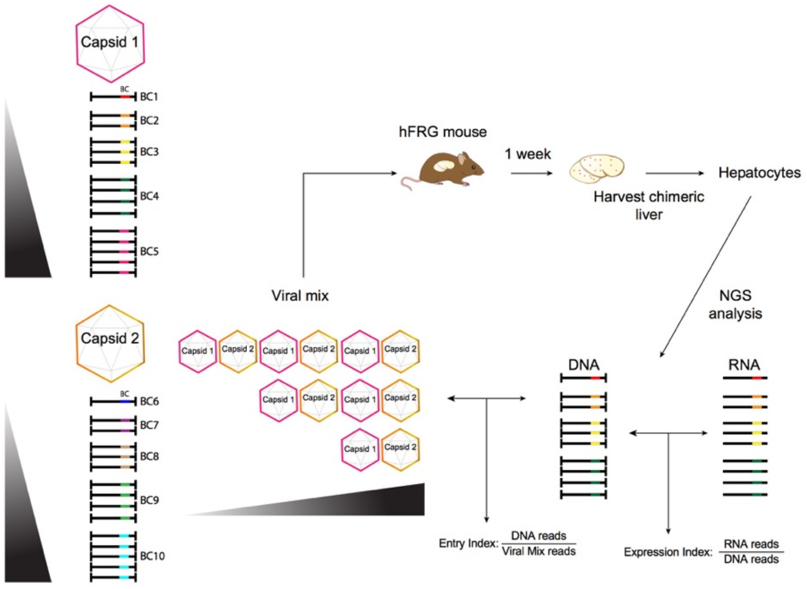

Supplementary Figure S5. Demultiplexed reads mapped to each cluster variant at Entry (A) and Expression (B) on human hepatocytes. Data pooled by cluster four origin can be found on Fig. 2D-E. (C) Entry index on murine cells sorted from the same hFRG. AAV2 variants are coloured according to Cluster 4 origin (aa 503, aa 596), yellow = AAV2 origin, salmon = AAV2.V59 origin.

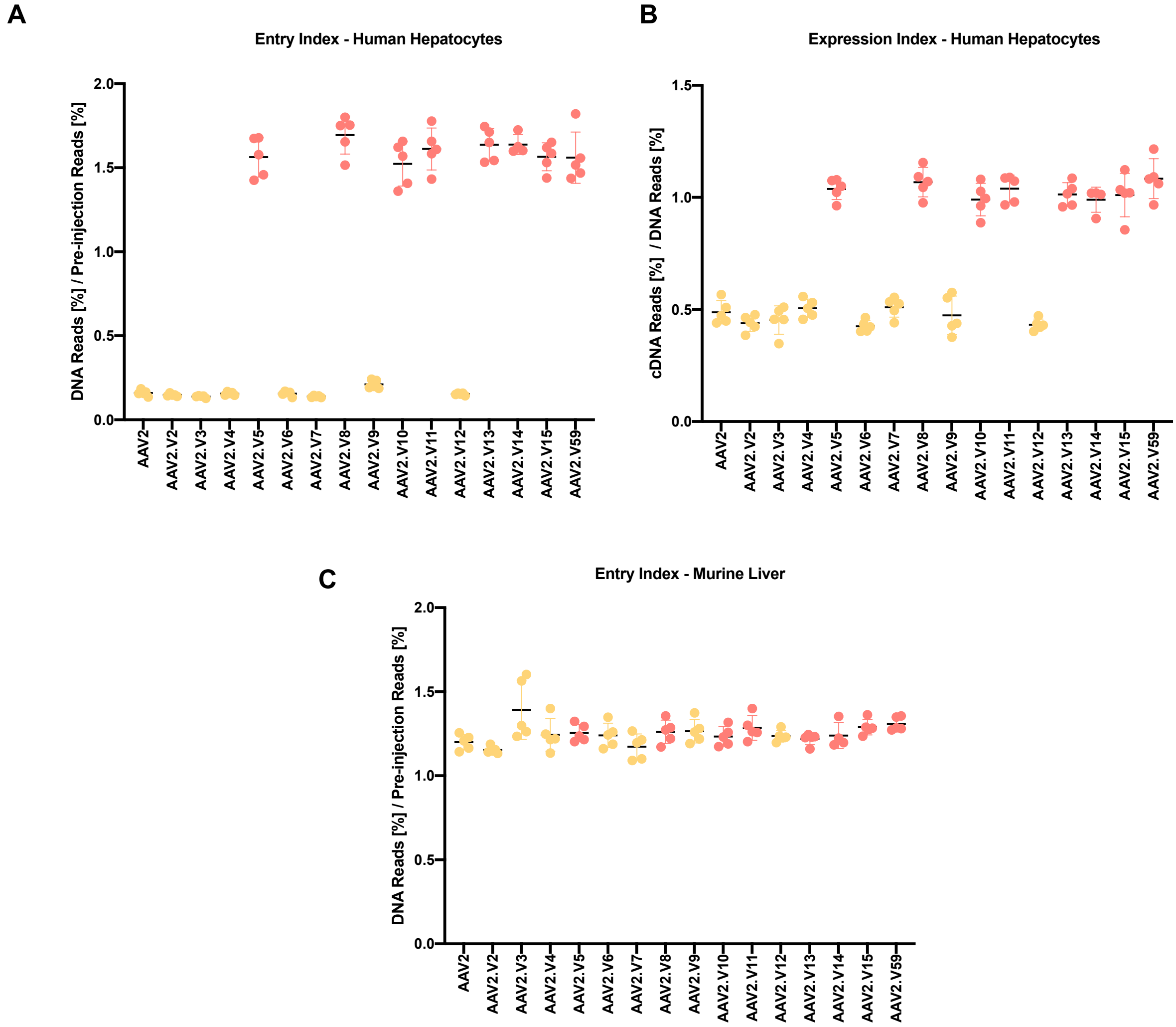

Supplementary Figure S6. (A) Relation between DNA read share (%) and RNA Read share (%) of listed AAV2-like variants harbouring T503 and N596 residues. (B) Similar relation of listed AAV2.V59-like variants, all harbouring A503 and D596 residues. The slope (0.4546 for AAV2-like and 1.046 for AAV2.V59-like variants) indicates relative vector transcription rate.

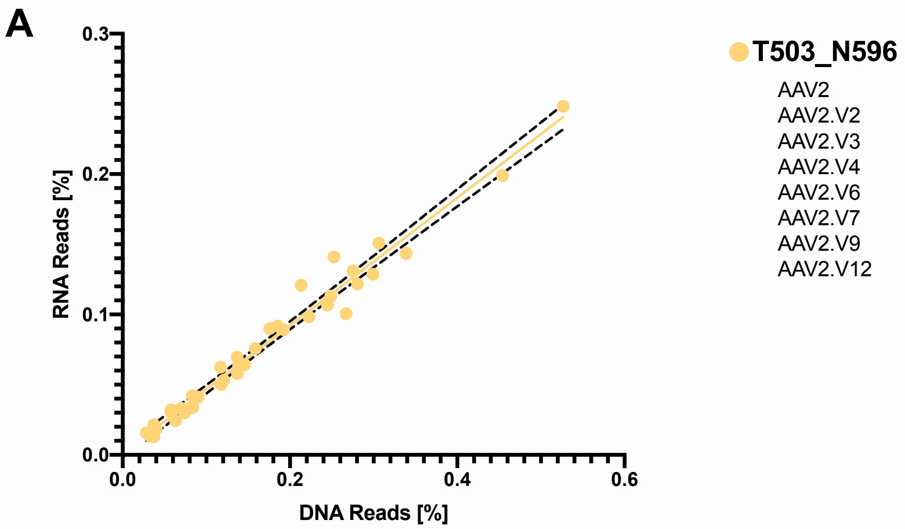

Equation	Y = 0.4546*X + 0.001244

R square	0.9769

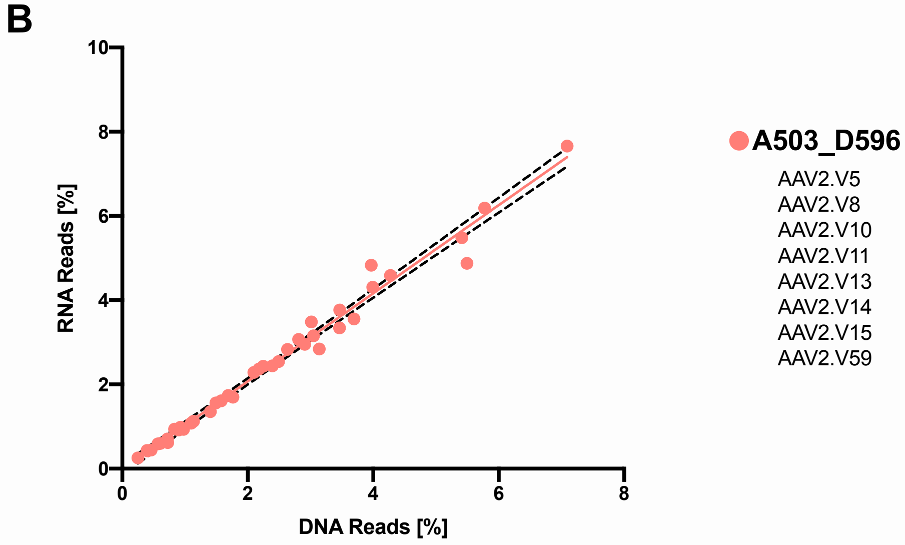

Equation	Y = 1.046*X - 0.02043

R square	0.9839

Supplementary Figure S7. HiTrap Heparin Column Binding assay of AAV2 Chromotography profile with the pooled fractions in the flow through (cyan) and elution (magenta) that were quantified by qPCR (A) and SDS-PAGE/Silver Staining of pre-loaded preparation (B) and fractions (C).

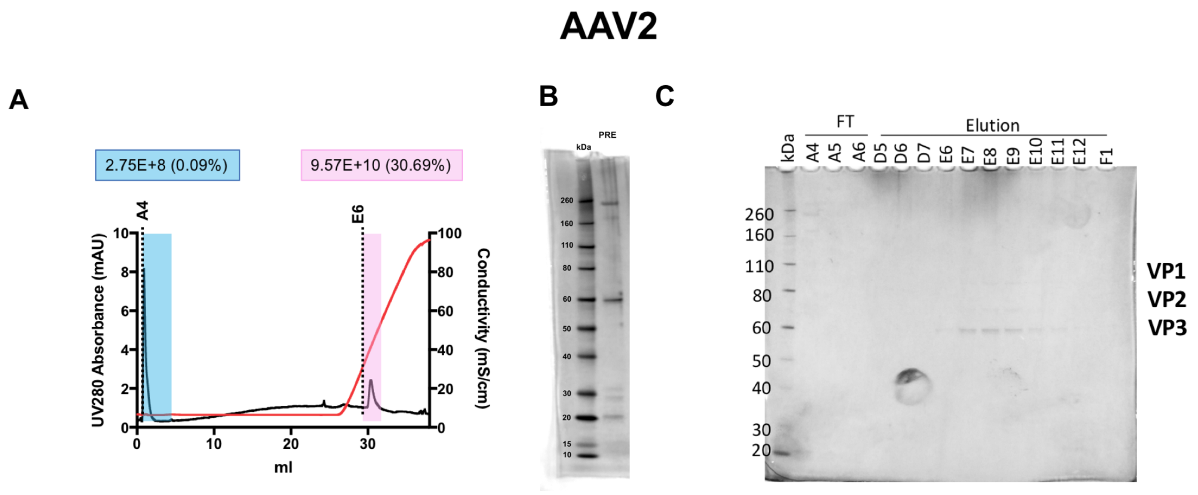

Supplementary Figure S8. HiTrap Heparin Column Binding assay of AAV2.V5 (a) Chromotography profile with the pooled fractions in the flow through (cyan) and elution (magenta) that were quantified by qPCR (a) and SDS-PAGE/Silver Staining (b).

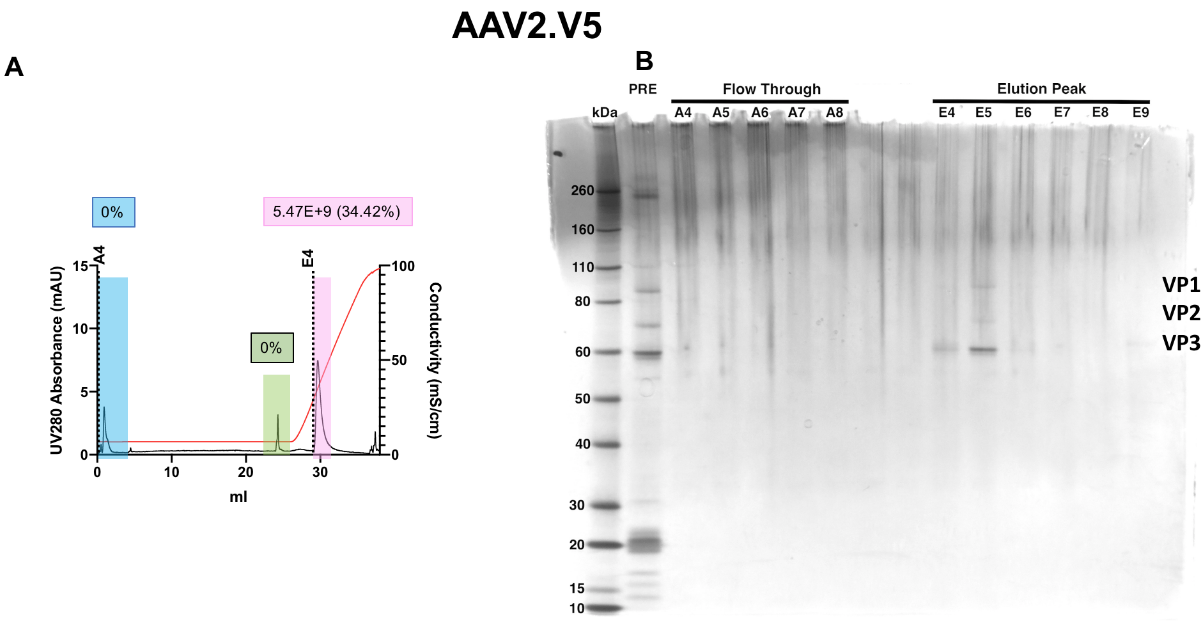

Supplementary Figure S9. HiTrap Heparin Column Binding assay of AAV2.V12 (a) Chromotography profile with the pooled fractions in the flow through (cyan) and elution magenta) that were quantified by qPCR (a) and SDS-PAGE/Silver Staining (b).

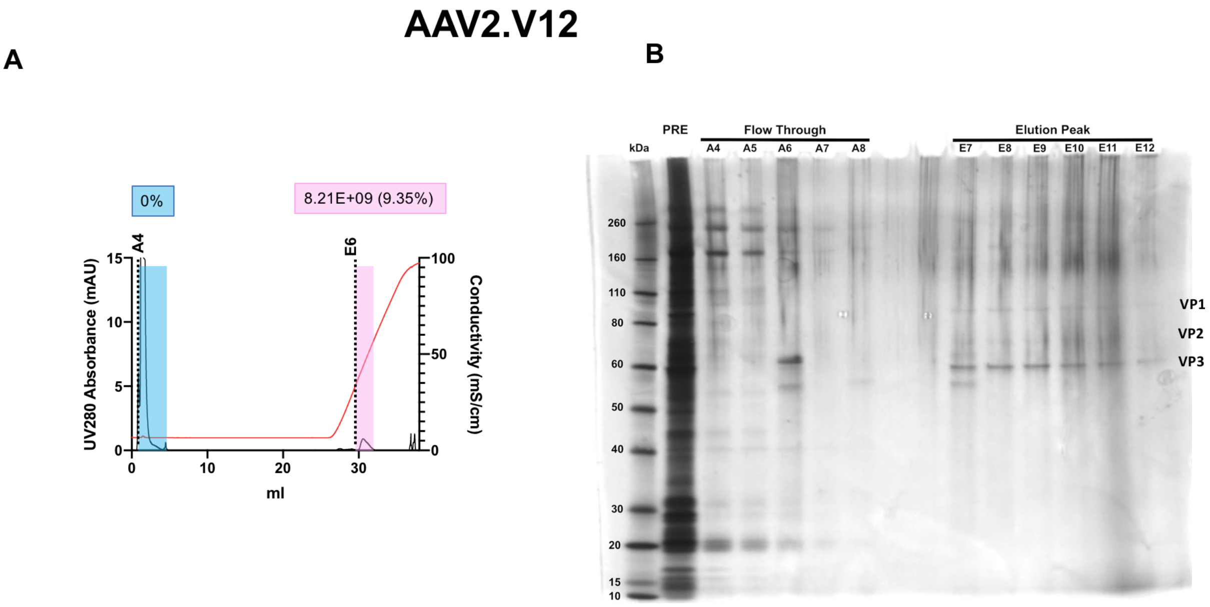

Supplementary Figure S10. HiTrap Heparin Column Binding assay of AAV2.V59 (a) Chromotography profile with the pooled fractions in the flow through (cyan) and elution magenta) that were quantified by qPCR (a) and SDS-PAGE/Silver Staining (b).

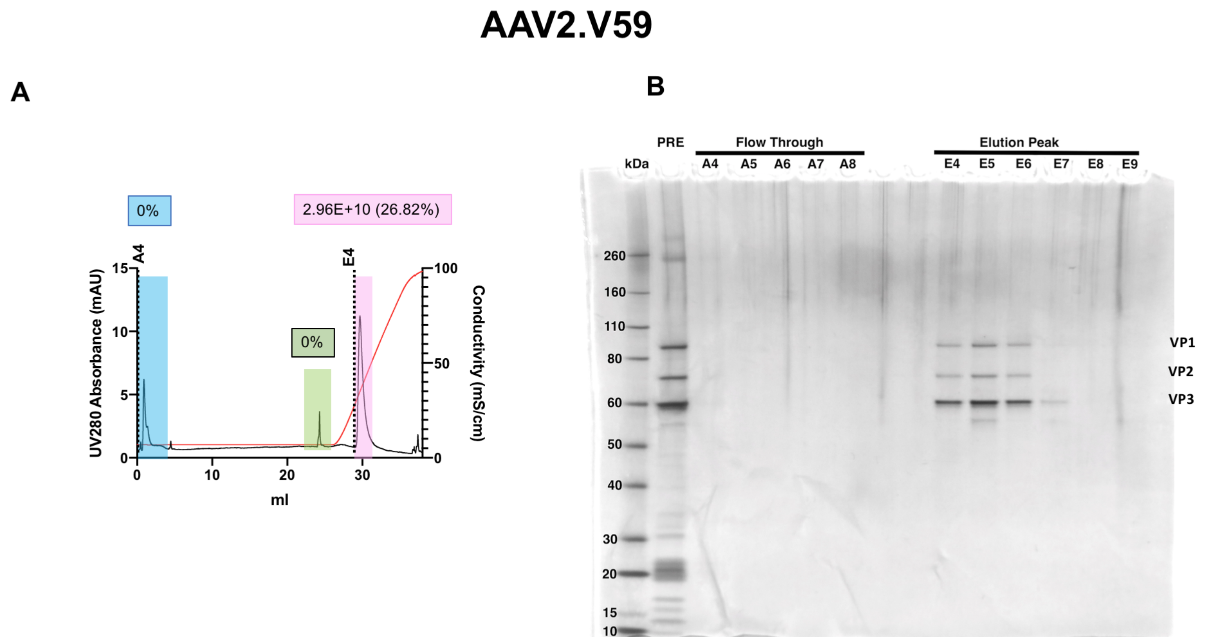

Supplementary Figure S11. HuH-7 transduction efficiency at 5,000 MOI (vg / cell) of n=8 AAV2-like clusters (harbouring amino acids T503 and N596) and n=8 AAV2.V59-like variants harbouring T503A and N596D mutations.

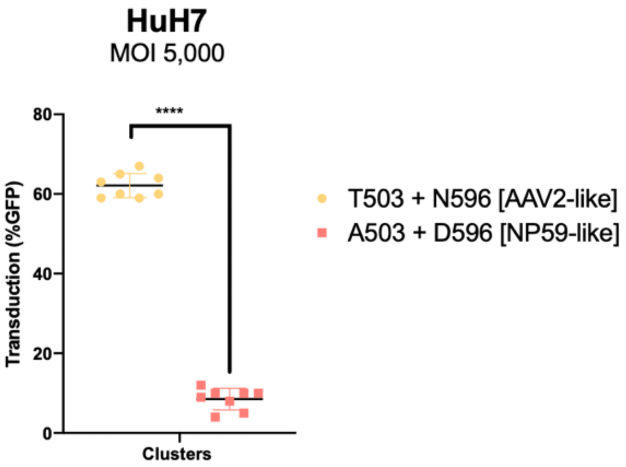

Supplementary Figure S12. *In vitro* heparin competition assay using HuH-7 cells transduced with AAV2, AAV2.V5, AAV2.V12 and AAV2.V59 expressing GFP, with and without soluble heparin (100 µg / mL, S12A-B, 400 µg / mL, S12C-D). Results expressed as a fold-reduction relative to the non-heparin condition at both percentage of GFP positive cells and mean fluorescence intensity (MFI).

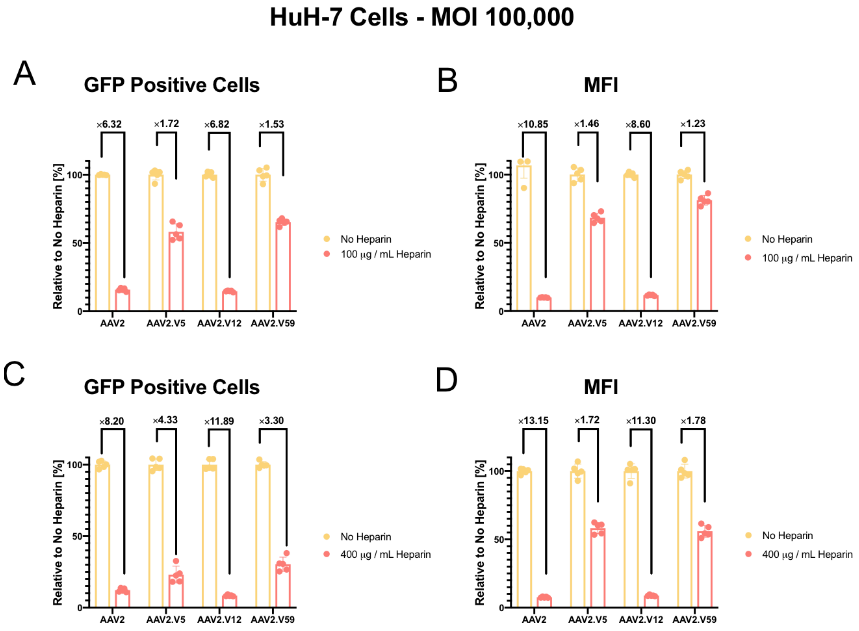

Supplementary Figure S13. Viral overlay assay. HuH-7 membrane proteins were incubated with indicated rAAV vectors at 5×10^{11} vg/mL. Membrane was then incubated with an anti-intact AAV2 A20 antibody and horseradish peroxidase (HRP)-conjugated secondary antibody was used then to detect signal. Membrane was then stripped and incubated with Anti-KIAA0319L (AAV-R) (Abcam, AB105385) at 1:400 dilution and signal was detected as described before.

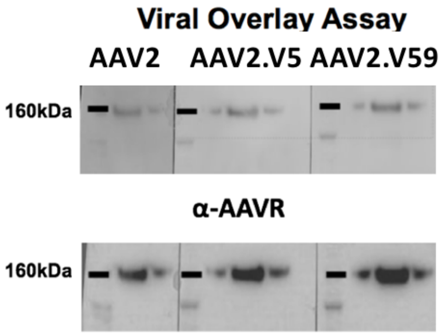

Supplementary Figure S14. AAV yield comparison from five independent crude productions using helper plasmids encoding for AAV2 variants V2-V15 and AAV2.V59. Vector yields are grouped by Cluster 4 origin (AAV2-like, T503 + N596; AAV-NP59 like, A503 + D596).

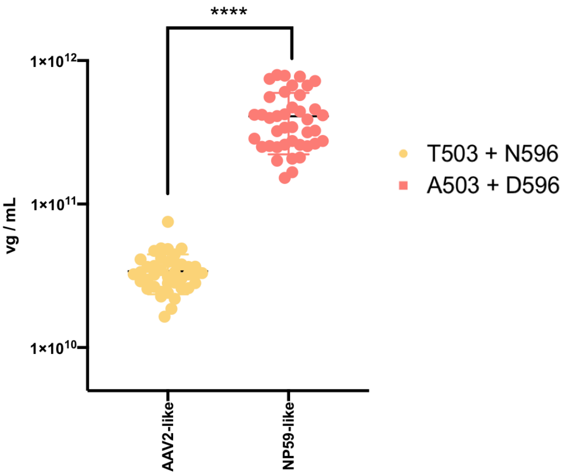

Supplementary Figure S15. (A) Next-generation sequencing reads (%) mapped to either the original AAV2 amino acid (yellow) or to the detected variant with increased representation at each especific AAV2 position (VP1 numbering). Frequency depicted for both the vector library and Round 4 of selection**. (B)** Entry index for sorted murine cells (DNA reads (%) / Pre-injection Reads (%).

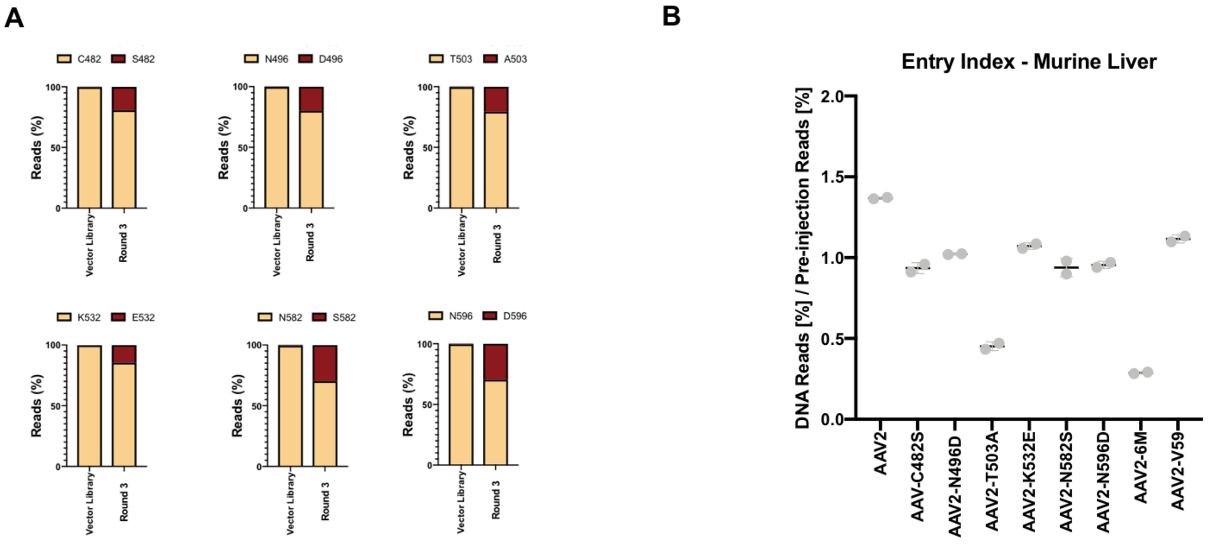

Supplementary Figure S16. HiTrap Heparin Column Binding assay of AAV2-N496D (a) Chromotography profile with the pooled fractions in the flow through (cyan) and elution magenta) that were quantified by qPCR (a) and SDS-PAGE/Silver Staining (b).

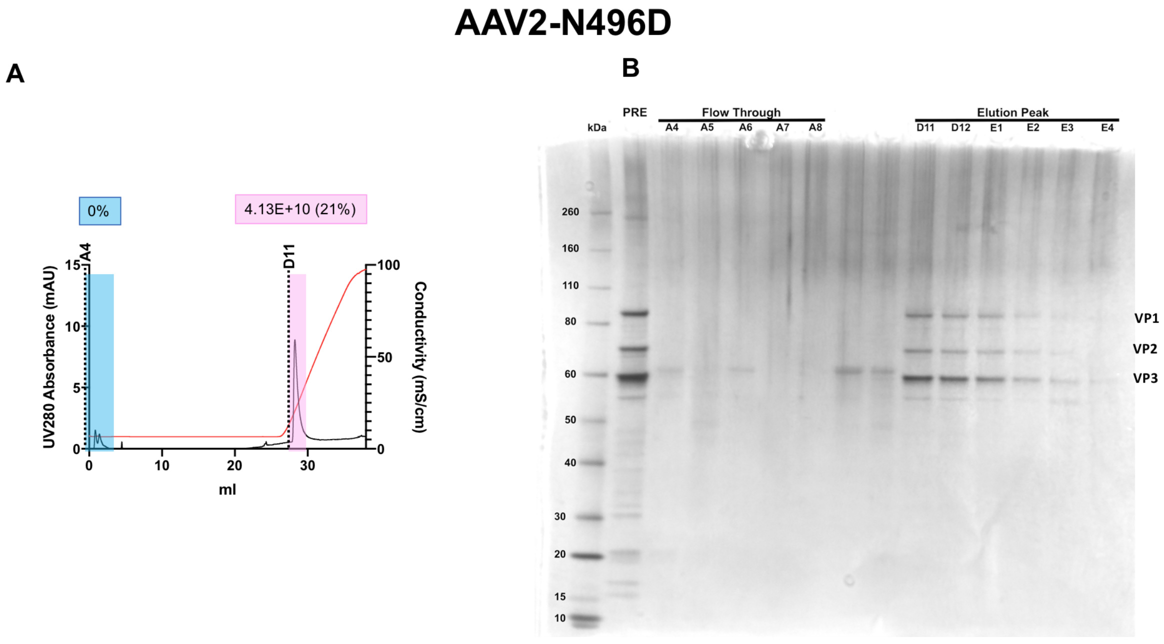

Supplementary Figure S17. HiTrap Heparin Column Binding assay of AAV2-N582S (a) Chromatography profile with the pooled fractions in the flow through (cyan) and elution magenta) that were quantified by qPCR (a) and SDS-PAGE/Silver Staining (b).

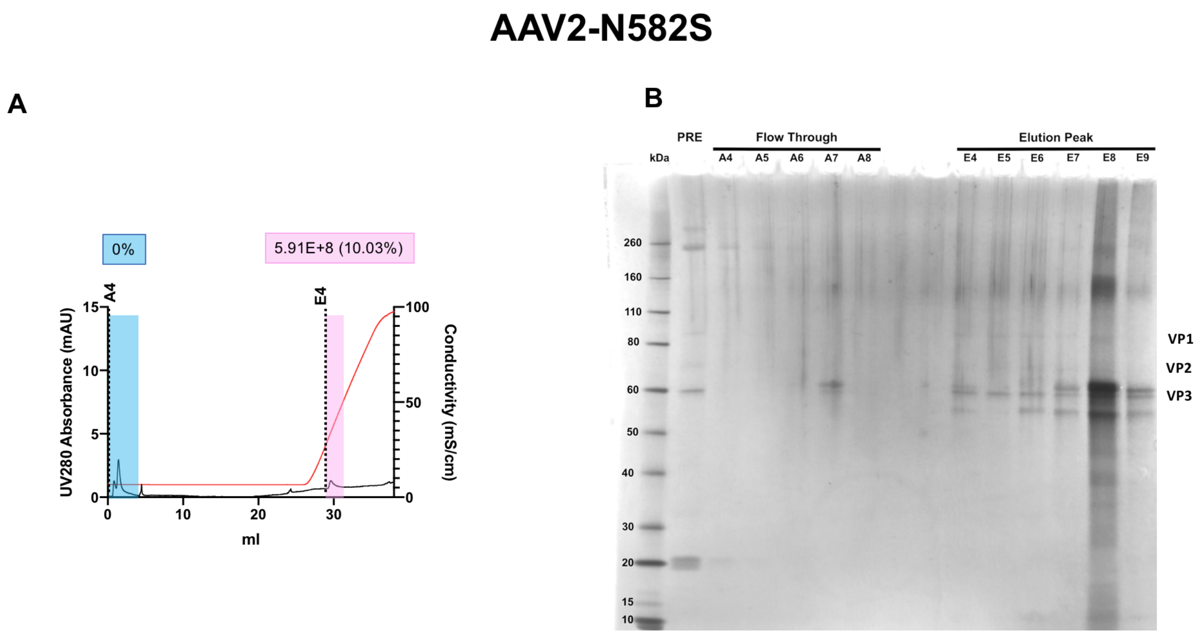

Supplementary Figure S18. HiTrap Heparin Column Binding assay of AAV2-K532E (a) Chromatography profile with the pooled fractions in the flow through (cyan) and elution magenta) that were quantified by qPCR (a) and SDS-PAGE/Silver Staining (b).

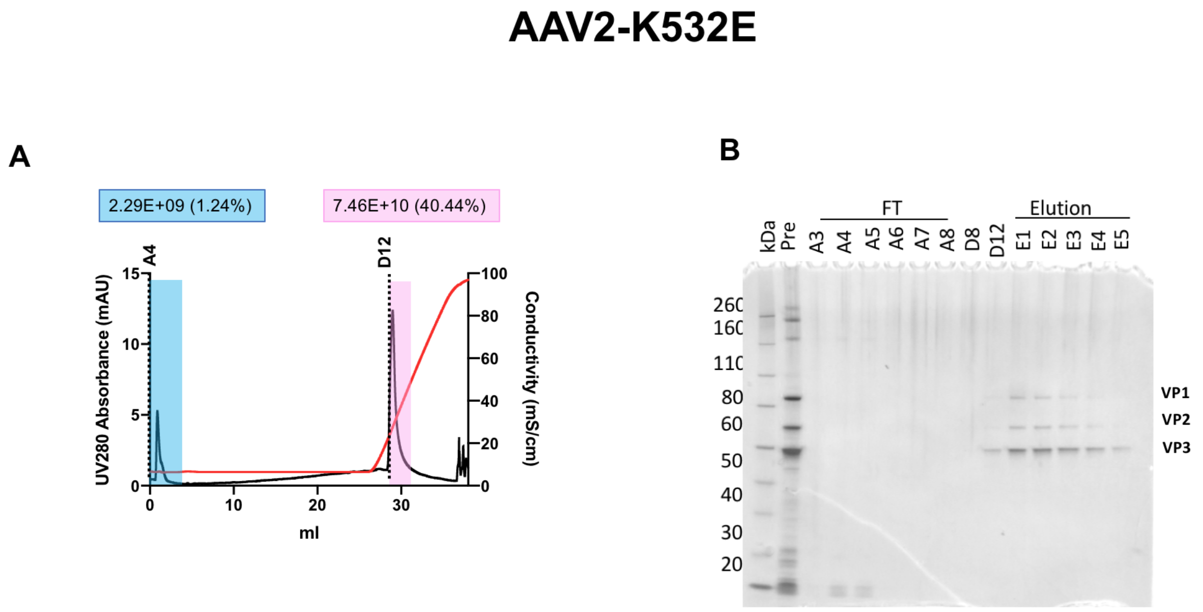

Supplementary Figure S19. HiTrap Heparin Column Binding assay of AAV2-6M (a) Chromotography profile with the pooled fractions in the flow through (cyan) and elution magenta) that were quantified by qPCR (a) and SDS-PAGE/Silver Staining (b).

AAV2-6M

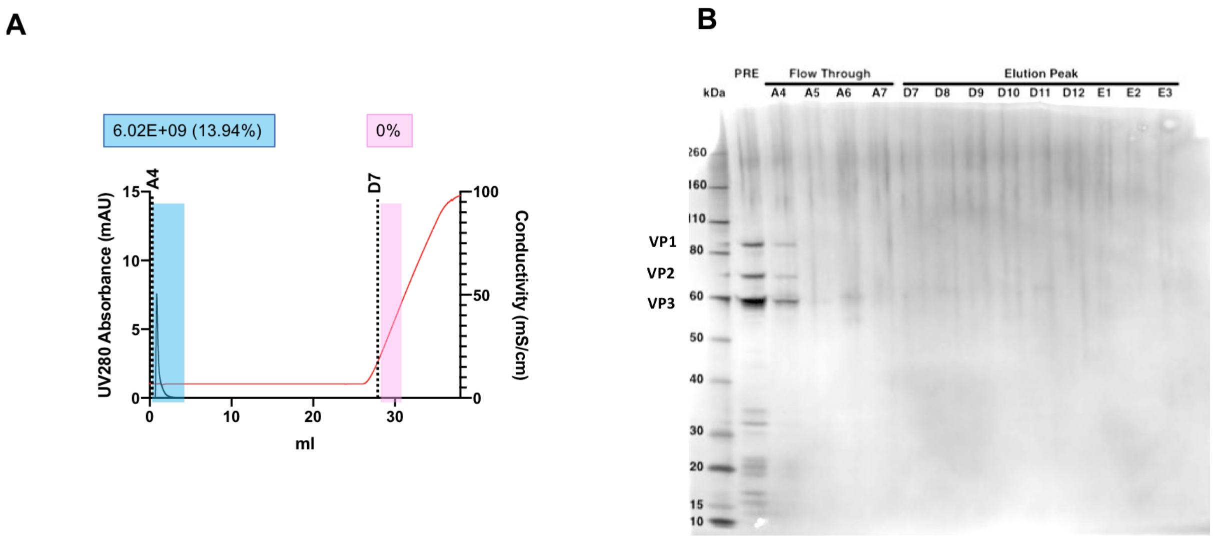

Supplementary Figure S20. HiTrap Heparin Column Binding assay of AAV8 (a) Chromotography profile with the pooled fractions in the flow through (cyan) and elution magenta) that were quantified by qPCR (a) and SDS-PAGE/Silver Staining (b).

AAV8

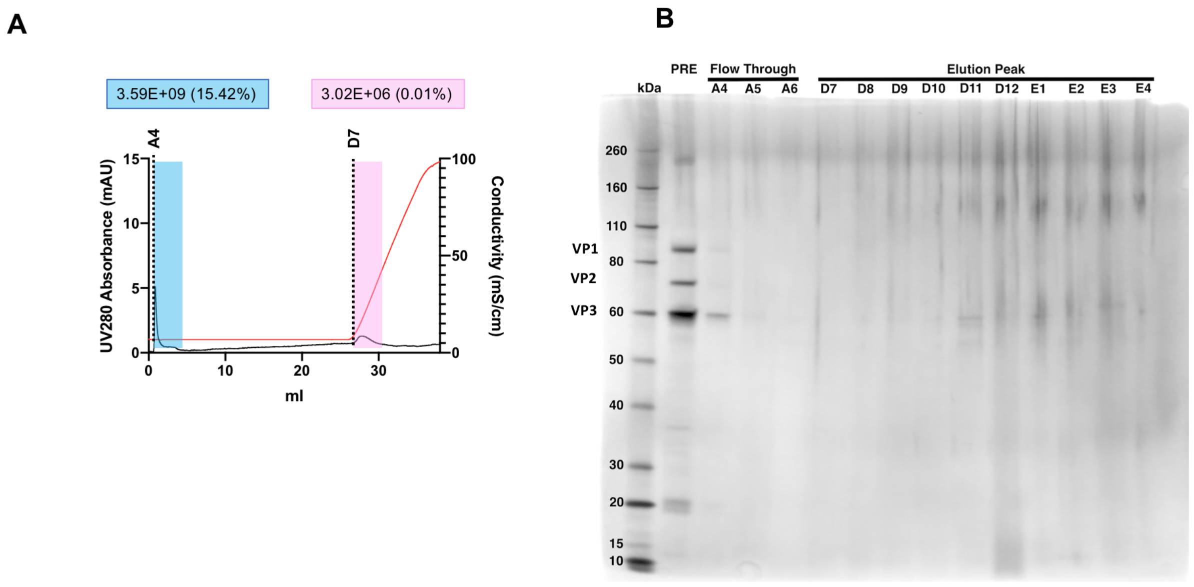

Supplementary Figure S21. HiTrap Heparin Column Binding assay of AAV8-E533K (a) Chromotography profile with the pooled fractions in the flow through (cyan) and elution magenta) that were quantified by qPCR (a) and SDS-PAGE/Silver Staining (b).

AAV8-E533K

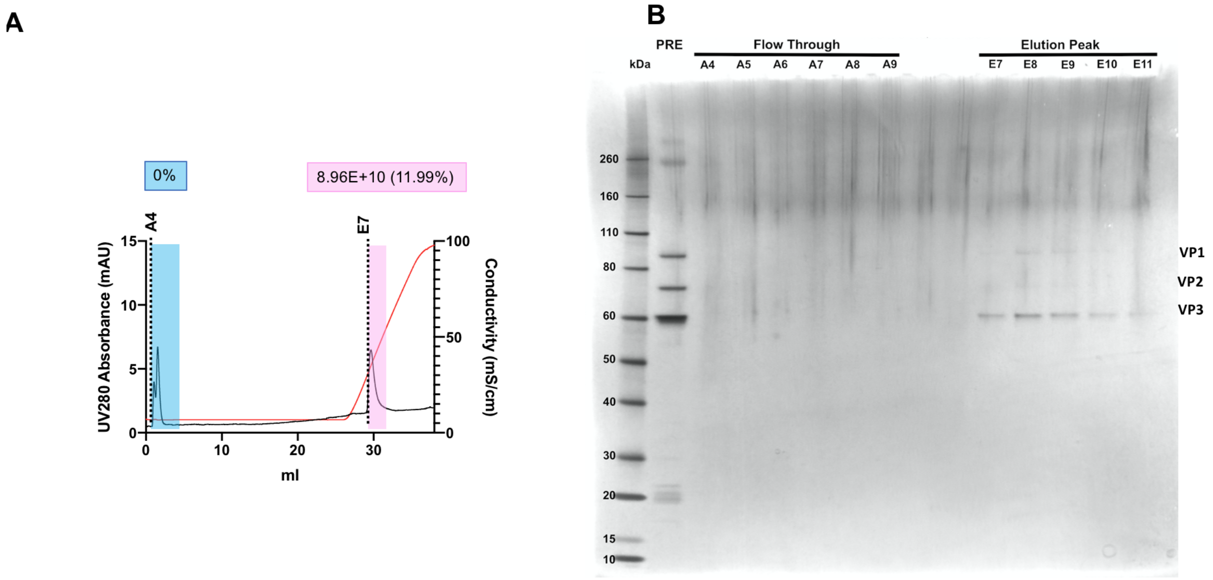

Supplementary Figure S22. HiTrap Heparin Column Binding assay of AAV8-RQNR (a) Chromotography profile with the pooled fractions in the flow through (cyan) and elution magenta) that were quantified by qPCR (a) and SDS-PAGE/Silver Staining (b).

AAV8-RQNR

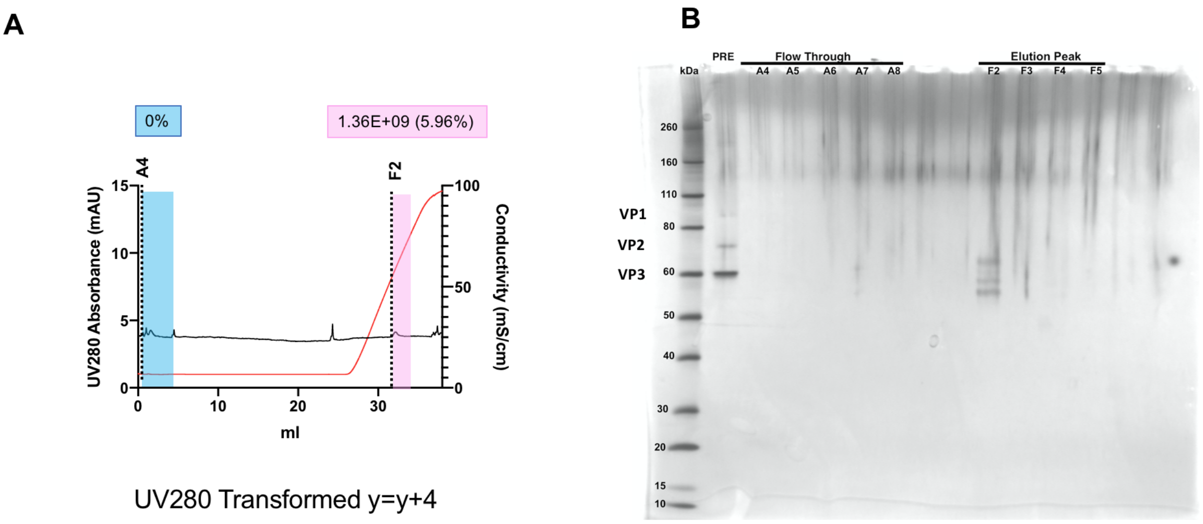

Supplementary Figure S23. HiTrap Heparin Column Binding assay of AAV8-E533K-N499D Chromatography profile with the pooled fractions in the flow through (cyan) and elution (magenta) that were quantified by qPCR (A) and SDS-PAGE/Silver Staining of pre-loaded preparation (B) and fractions (C).

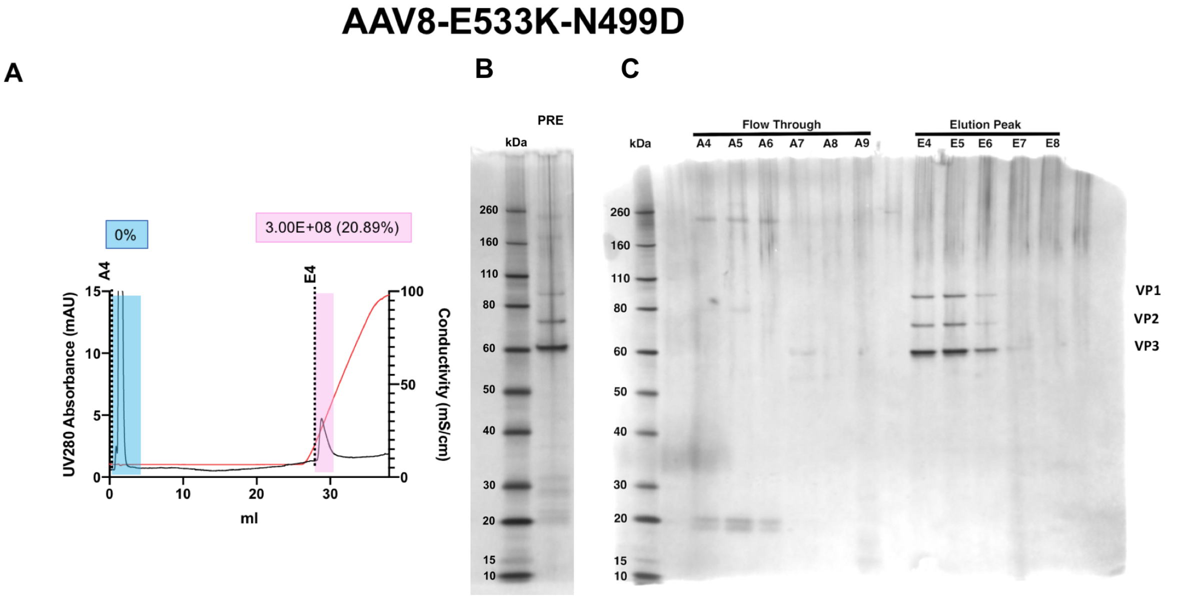

Supplementary Figure S24. HiTrap Heparin Column Binding assay of AAV8-RQNR-N499D (a) Chromatography profile with the pooled fractions in the flow through (cyan) and elution magenta) that were quantified by qPCR (a) and SDS-PAGE/Silver Staining (b).

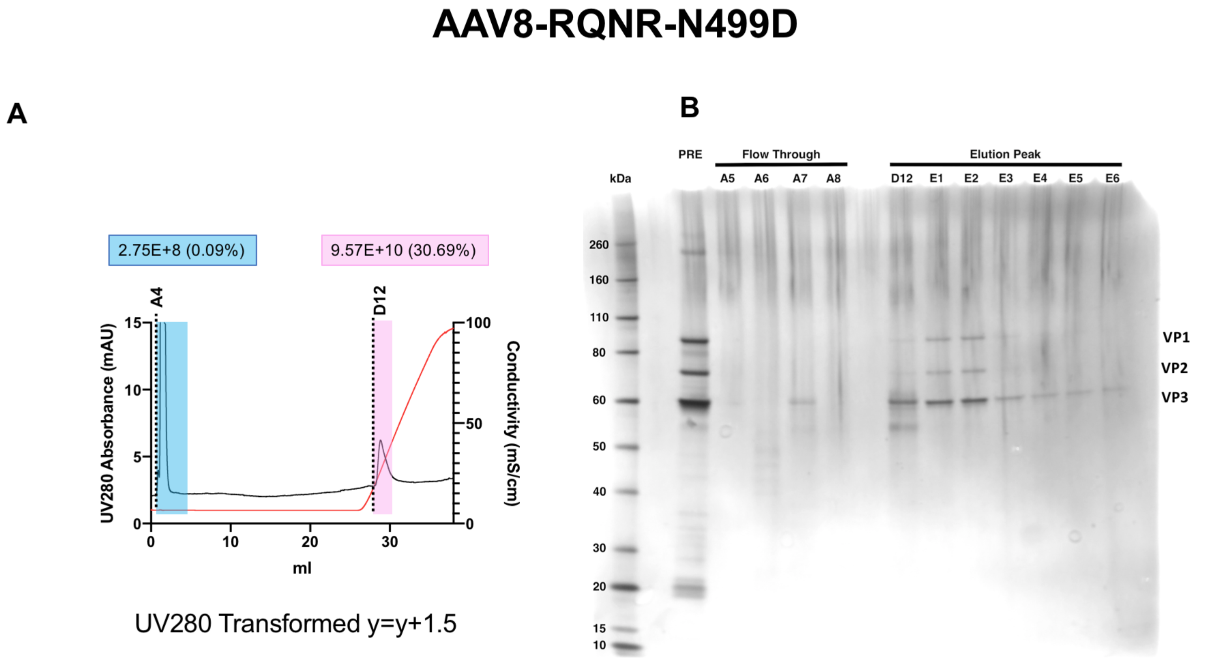

Supplementary Figure S25. AAV yield comparison from three independent crude preparations using helper plasmids encoding for AAV8, AAV8-RQNR, AAV8-RQNR-N499D, AAV8-E533K and AAV8-E533K-N499D.

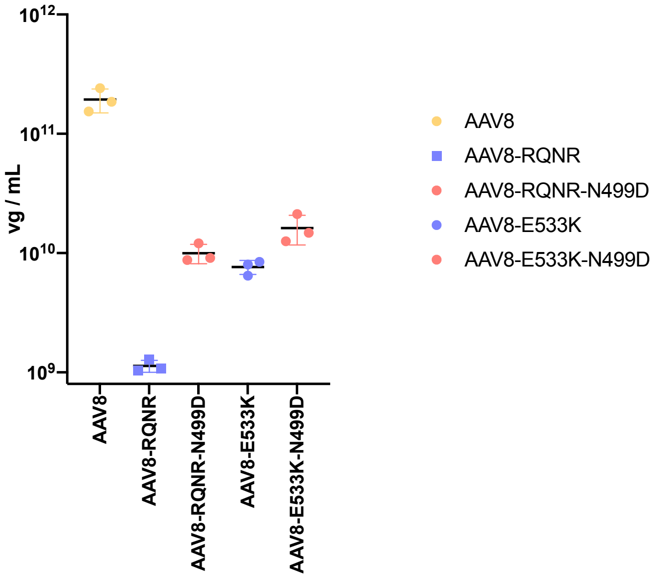

Supplementary Figure S26. Gibson Assembly based Construction of library AAV2[Lib2048.] Four AAV2 backbone variants encoding for the full cap ORF were first generated (prototypical AAV2, a T503A variant, a N596D variant and a double mutant). The DNA fragments englobing all the possible combinations corresponding to the first five mutations between AAV2 and NP59 were custom synthesized (2^5 = 32 fragments), as well as the following four (2^4 = 16 fragments). Fragments were individually PCR amplified with overlapping primers and Gibson assembled on an equimolar ratio to the PCR amplified and DpnI treated backbones englobing the four distinct variants (2^2 = 4). Thus, the total complexity of the library was expected to be of 2048 variants (32 * 16 * 4), equivalent to the permutation of the eleven variable amino acids (2^{11} = 2048).

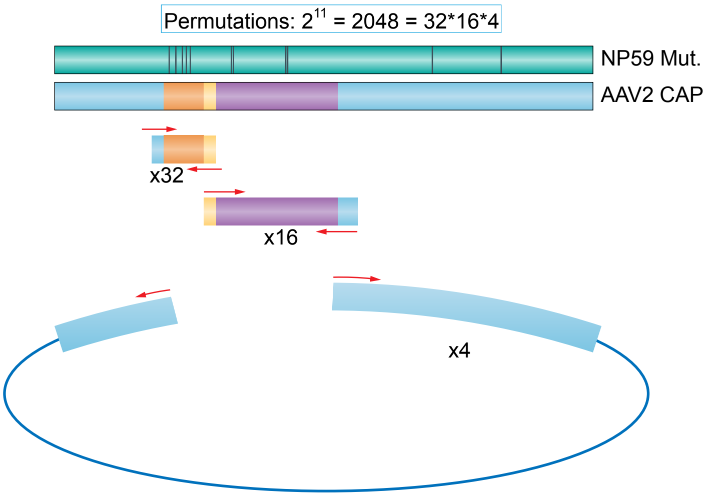

Supplementary Table ST1. Eleven amino acid differences between AAV2 and AAV-NP59, corresponding origin on the initial shuffled library and corresponding structural cluster (1-4).

Mutation #	Position	AAV2 Amino acid	NP59 Amino acid	Probable Origin	Structural Cluster
1	162	A	T	AAV1/AAV6	1
2	168	R	K	AAV1/AAV6	1
3	179	A	S	AAV1/AAV6	1
4	180	D	E	AAV1/AAV6	1
5	190	Q	E	AAV3	1
6	233	T	Q	AAV3	2
7	235	M	L	AAV3	2
8	310	R	K	AAV3	3
9	312	N	S	AAV3	3
10	503	T	A	Point Mutation	4
11	596	N	D	Point Mutation	4

Supplementary Table ST2. Raw viral mix NGS read counts and percentage (%) mapped to each capsid (n=5 BC / capsid) at increasing concentration. For variant AAV2.V14, barcode A was disregarded for further analysis due to significantly lower concentration than barcode A population average (thus n=4 BC / capsid for AAV2.V14).

AAV2_A	4161	0.24	2_2A	5078	0.29	2_3A	4518	0.26	2_3A	4518	0.26	2_4A	4448	0.25	2_5A	3061	0.17
AAV2_B	8679	0.49	2_2B	7924	0.45	2_3B	9500	0.54	2_3B	9500	0.54	2_4B	9018	0.51	2_5B	9104	0.52
AAV2_C	17341	0.98	2_2C	20282	1.15	2_3C	19311	1.10	2_3C	19311	1.10	2_4C	18484	1.05	2_5C	17610	1.00
AAV2_D	17434	0.99	2_2D	30775	1.75	2_3D	28094	1.60	2_3D	28094	1.60	2_4D	26339	1.50	2_5D	28760	1.63
AAV2_E	25698	1.46	2_2E	42042	2.39	2_3E	36951	2.10	2_3E	36951	2.10	2_4E	30108	1.71	2_5E	32654	1.85
2_6A	4242	0.24	2_7A	2684	0.15	2_8A	4764	0.27	2_9A	6705	0.38	2_10A	4500	0.26	2_11A	7286	0.41
2_6B	9176	0.52	2_7B	4228	0.24	2_8B	10548	0.60	2_9B	13140	0.75	2_10B	11134	0.63	2_11B	15941	0.91
2_6C	18334	1.04	2_7C	10415	0.59	2_8C	13622	0.77	2_9C	33071	1.88	2_10C	18640	1.06	2_11C	35165	2.00
2_6D	25535	1.45	2_7D	9140	0.52	2_8D	27627	1.57	2_9D	53188	3.02	2_10D	31936	1.81	2_11D	27591	1.57
2_6E	34205	1.94	2_7E	17101	0.97	2_8E	46294	2.63	2_9E	58798	3.34	2_10E	42057	2.39	2_11E	58246	3.31
2_12A	4868	0.28	2_13A	9094	0.52	2_14A	5	0.00	2_15A	5290	0.30	2_59A	5994	0.34			
2_12B	8783	0.50	2_13B	21980	1.25	2_14B	12086	0.69	2_15B	11179	0.63	2_16B	12547	0.71			
2_12C	8975	0.51	2_13C	40250	2.29	2_14C	25891	1.47	2_15C	22072	1.25	2_16C	24505	1.39			
2_12D	27570	1.57	2_13D	58625	3.33	2_14D	36974	2.10	2_15D	31147	1.77	2_16D	34615	1.97			
2_12E	28731	1.63	2_13E	79892	4.54	2_14E	42509	2.41	2_15E	35734	2.03	2_16E	58873	3.34			

Supplementary Table ST3. Primers used for site-directed mutagenesis of corresponding AAV variants.

Variant	AAV2 T503A	Sequence
Forward	F_AAV2_T503A	TACTCGTGGGCTGGAGCTACCAAGTAC
Reverse	R_AAV2_T503A	TTCACTGTTGTTGTTATCCGCAGATG
**Variant**	**AAV2 N596D**	**Sequence**
Forward	F_AAV2_N596D	CGCAGATGTCAACACACAAGGCG
Reverse	R_AAV2_N596D	GTAGCTGCTTGTCTGTTGCCTCTCT
**Variant**	**AAV2 C482S**	**Sequence**
Forward	F_AAV2_C482S	GGCTTCCTGGACCCAGTTACCGCCAGCAG
Reverse	R_AAV2_C482S	AGTTCCTAGACTGGTCCCGAATGTC
**Variant**	**AAV2 N496D**	**Sequence**
Forward	F_AAV2_N496D	CATCTGCGGATAACGACAACAGTGAATACTC
Reverse	R_AAV2_N496D	TCTTTGATACTCGCTGCTGGCG
**Variant**	**AAV2 K532E**	**Sequence**
Forward	F_AAV2_K532E	GGACGATGAAGAAGAGTTTTTTCCTCAGAGCG
Reverse	R_AAV2_K532E	TTGTGGCTTGCCATGGCCG
**Variant**	**AAV2 N582S**	**Sequence**
Forward	F_AAV2_N582S	GTTCTGTATCTACCAGCCTCCAGAGAGGCAACAG
Reverse	R_AAV2_N582S	CATACTGCTCCGTAGCCACGGG
**Variant**	**AAV8 E533K**	**Sequence**
Forward	F_AAV8_E533K	ACACAAAGACGACAAGGAGCGTTTTTTTCCCAG
Reverse	R_AAV8_E533K	GTTGCCATAGCGATGCCAGGATTAGCC
**Variant**	**AAV8 RQNR**	**Sequence**
Forward	F_AAV8_RQNR	GGCAGATAACTTGCAGAGGCAAAACAGGGCTCCTCAAATTGG
Reverse	R_AAV8_RQNR	ACGATACCGTATTCCTCTGTAGCCAC
**Variant**	**AAV8 E533K/RQNR + N499D**	**Sequence**
Forward	F_AAV8_N499D	CGACAACCGGGCAAAACGACAATAGCAACTTTGCC
Reverse	R_AAV8_N499D	TTGAGACGCGTTGTTGGCGGTAACAGG

Supplementary Table ST4. Primers.

Primer Name	Sequence
GFP-qPCR-For	TCAAGATCCGCCACAACATC
GFP-qPCR-Rev	TTCTCGTTGGGGTCTTTGCT
BC_F	GCTGGAGTTCGTGACCGCCG
BC_R	CAACATAGTTAAGAATACCAGTCAATCTTTCACAAATTTTGTAATCCAGAGG
human ALB_F ddPCR	TGCTGTCATCTCTTGTGGGCTG
human ALB_R ddPCR	AACTCATGGGAGCTGCTGGTTC
Cluster1_F	TGTGGAGCCAGACTCCTCCT
Cluster1_R	GTTCCCAGACCAGAGGGGG
Cluster2_F	CACCAATGGCAGACAATAACGAGGG
Cluster2_R	GTTTGTAGAGGTGGTTGTTGTAGGTGG
Cluster3_F	ATTCCACTGCCACTTTTCACCAC
Cluster3_R	AGGTTATTGGCAATCGTCGTCGT
Cluster4_F	TCTAGGAACTGGCTTCCTGGACCC
Cluster4_R	CAGATGGGCCCCTGAAGGTACACATC
CAP Rescue F	CCCTGCAGACAATGCGAGAGAATGAATCAGAATTCAAATATCTGC
CAP Rescue R	ATGCATATGGAAACTAG ATAAGAAAGAAATACG

Supplementary Table ST5. FRG IDs and related information.

FRG ID	Gender	Age at injection (weeks)	[hAlbumin] at injection (mg/mL)	Experiment	Figure
203	female	24	4.55	AAV2, AAV-NP59, AAV2.V59 comparison	1A
38	female	24	7.8	Round 1 - FT-2048 Library Selection	/
58	female	24	6.45	Round 2 - FT-2048 Library Selection	1D
316	female	24	2.64	Round 3 - FT-2048 Library Selection	/
235	female	24	6.72	Round 4 - FT-2048 Library Selection	1E-F
605	female	20	0.82	AAV2 Clusters - Variants Comparison	2C-D
21	female	24	3.22	AAV2, AAV2.V59, AAV2.V5 Comparison	2E
36	female	24	8	Round 1 - FT Library NP59	/
56	female	24	8.7	Round 2 - FT Library NP59	/
234	female	24	8.55	Round 3 - FT Library NP59	SF 15
303	female	28	0.53	AAV2 Point Mutation Variants Comparison	3C-D
87	female	24	0.204	AAV2 IHC	3E-F
146	female	24	0.541	AAV2.V59 IHC	3E-F
29	female	24	0.2325	AAV2-N496D IHC	3E-F
128	female	24	0.571	AAV2-N582S IHC	3E-F
225	male	20	non-engrafted	AAV8 IHC	4A
226	male	20	non-engrafted	AAV8-E533K IHC	4A
227	male	20	non-engrafted	AAV8-RQNR IHC	4A
272	male	22	non-engrafted	AAV8 LSP Variants NGS Comparison	4B
216	male	24	non-engrafted	AAV8 IHC Low Dose	4C
600	male	23	non-engrafted	AAV8 IHC High Dose	4C
602	male	23	non-engrafted	AAV8-E533K IHC	4C
603	male	23	non-engrafted	AAV8-RQNR IHC	4C
608	male	23	non-engrafted	AAV8-E533-N499D IHC	4C
607	male	23	non-engrafted	AAV8-RQNR-N499D IHC	4C

Statistical analysis

Fig. 2E	Entry Index
Column B	A503 + D596 [AAV2_V59-like]
vs.	vs.
Column A	T503 + N596 [AAV2-like]
Mann Whitney test	
P value	<0.0001
Exact or approximate P value?	Exact
P value summary	****
Significantly different (P < 0.05)?	Yes
One- or two-tailed P value?	Two-tailed
Sum of ranks in column A,B	820 , 2420
Difference between medians	
Median of column A	0.1516, n=40
Median of column B	1.606, n=39
Difference: Actual	1.454
Difference: Hodges-Lehmann	1.45

Fig. 2F	Expression Index
Column B	A503 + D596 [AAV2_V59-like]
vs.	vs.
Column A	T503 + N596 [AAV2-like]
Mann Whitney test	
P value	<0.0001
Exact or approximate P value?	Exact
P value summary	****
Significantly different (P < 0.05)?	Yes
One- or two-tailed P value?	Two-tailed
Sum of ranks in column A,B	820 , 2420
Difference between medians	
Median of column A	0.4561, n=40
Median of column B	1.025, n=39
Difference: Actual	0.5686
Difference: Hodges-Lehmann	0.5647

Fig. 2G	
Column B	AAV2.V59 DNA
vs.	vs.
Column A	AAV2 DNA
Mann Whitney test	
P value	<0.0001
Exact or approximate P value?	Exact
P value summary	****
Significantly different (P < 0.05)?	Yes
One- or two-tailed P value?	Two-tailed
Sum of ranks in column A,B	45 , 126
Difference between medians	
Median of column A	0.5714, n=9
Median of column B	5.078, n=9
Difference: Actual	4.507
Difference: Hodges-Lehmann	4.451

Fig. 2G	
Column B	AAV2.V5 DNA
vs.	vs.
Column A	AAV2 DNA
Mann Whitney test	
P value	<0.0001
Exact or approximate P value?	Exact
P value summary	****
Significantly different (P < 0.05)?	Yes
One- or two-tailed P value?	Two-tailed
Sum of ranks in column A,B	45 , 126
Difference between medians	
Median of column A	0.5714, n=9
Median of column B	4.832, n=9
Difference: Actual	4.26
Difference: Hodges-Lehmann	4.253

Fig. 2G	
Column B	AAV2.V5 DNA
vs.	vs.
Column A	AAV2.V59 DNA
Mann Whitney test	
P value	0.3865
Exact or approximate P value?	Exact
P value summary	ns
Significantly different (P < 0.05)?	No
One- or two-tailed P value?	Two-tailed
Sum of ranks in column A,B	96 , 75
Difference between medians	
Median of column A	5.078, n=9
Median of column B	4.832, n=9
Difference: Actual	-0.2468
Difference: Hodges-Lehmann	-0.1764

Fig. 2G	
Column B	AAV2.V59 cDNA
vs.	vs.
Column A	AAV2 cDNA
Mann Whitney test	
P value	<0.0001
Exact or approximate P value?	Exact
P value summary	****
Significantly different (P < 0.05)?	Yes
One- or two-tailed P value?	Two-tailed
Sum of ranks in column A,B	45 , 126
Difference between medians	
Median of column A	0.1585, n=9
Median of column B	5.217, n=9
Difference: Actual	5.059
Difference: Hodges-Lehmann	5.061

Fig. 2G	
Column B	AAV2.V5 cDNA
vs.	vs.
Column A	AAV2 cDNA
Mann Whitney test	
P value	<0.0001
Exact or approximate P value?	Exact
P value summary	****
Significantly different (P < 0.05)?	Yes
One- or two-tailed P value?	Two-tailed
Sum of ranks in column A,B	45 , 126
Difference between medians	
Median of column A	0.1585, n=9
Median of column B	5.044, n=9
Difference: Actual	4.886
Difference: Hodges-Lehmann	4.886

Fig. 2G	
Column B	AAV2.V59 cDNA
vs.	vs.
Column A	AAV2.V5 cDNA
Mann Whitney test	
P value	0.6048
Exact or approximate P value?	Exact
P value summary	ns
Significantly different (P < 0.05)?	No
One- or two-tailed P value?	Two-tailed
Sum of ranks in column A,B	79 , 92
Difference between medians	
Median of column A	5.044, n=9
Median of column B	5.217, n=9
Difference: Actual	0.173
Difference: Hodges-Lehmann	0.1687

Fig. 3G	
Column B	N496D
vs.	vs.
Column A	AAV2
Mann Whitney test	
P value	<0.0001
Exact or approximate P value?	Exact
P value summary	****
Significantly different (P < 0.05)?	Yes
One- or two-tailed P value?	Two-tailed
Sum of ranks in column A,B	55 , 155
Mann-Whitney U	0
Difference between medians	
Median of column A	2.305, n=10
Median of column B	56.50, n=10
Difference: Actual	54.2
Difference: Hodges-Lehmann	54.1

Fig. 3G	
Column B	N582S
vs.	vs.
Column A	AAV2
Mann Whitney test	
P value	<0.0001
Exact or approximate P value?	Exact
P value summary	****
Significantly different (P < 0.05)?	Yes
One- or two-tailed P value?	Two-tailed
Sum of ranks in column A,B	55 , 155
Mann-Whitney U	0
Difference between medians	
Median of column A	2.305, n=10
Median of column B	52.50, n=10
Difference: Actual	50.2
Difference: Hodges-Lehmann	50.12

Fig. 3G	
Column B	AAV2.V59
vs.	vs.
Column A	AAV2
Mann Whitney test	
P value	<0.0001
Exact or approximate P value?	Exact
P value summary	****
Significantly different (P < 0.05)?	Yes
One- or two-tailed P value?	Two-tailed
Sum of ranks in column A,B	55 , 155
Mann-Whitney U	0
Difference between medians	
Median of column A	2.305, n=10
Median of column B	62.00, n=10
Difference: Actual	59.7
Difference: Hodges-Lehmann	59.7

Fig. 3G	
Column B	N496D
vs.	vs.
Column A	N582S
Mann Whitney test	
P value	0.9831
Exact or approximate P value?	Exact
P value summary	ns
Significantly different (P < 0.05)?	No
One- or two-tailed P value?	Two-tailed
Sum of ranks in column A,B	104.5 , 105.5
Difference between medians	
Median of column A	52.50, n=10
Median of column B	56.50, n=10
Difference: Actual	4
Difference: Hodges-Lehmann	0.5

Fig. 3G	
Column B	N496D
vs.	vs.
Column A	AAV2.V59
Mann Whitney test	
P value	0.1008
Exact or approximate P value?	Exact
P value summary	ns
Significantly different (P < 0.05)?	No
One- or two-tailed P value?	Two-tailed
Sum of ranks in column A,B	127 , 83
Difference between medians	
Median of column A	62.00, n=10
Median of column B	56.50, n=10
Difference: Actual	-5.5
Difference: Hodges-Lehmann	-8

Fig. 3G	
Column B	N582S
vs.	vs.
Column A	AAV2.V59
Mann Whitney test	
P value	0.1372
Exact or approximate P value?	Exact
P value summary	ns
Significantly different (P < 0.05)?	No
One- or two-tailed P value?	Two-tailed
Sum of ranks in column A,B	125 , 85
Difference between medians	
Median of column A	62.00, n=10
Median of column B	52.50, n=10
Difference: Actual	-9.5
Difference: Hodges-Lehmann	-9

Column B	AAV-NP59
vs.	vs.
Column A	AAV2
Mann Whitney test	
P value	0.0079
Exact or approximate P value?	Exact
P value summary	**
Significantly different (P < 0.05)?	Yes
One- or two-tailed P value?	Two-tailed
Sum of ranks in column A,B	15 , 40
Difference between medians	
Median of column A	24497162657, n=5
Median of column B	419477000000, n=5
Difference: Actual	3.9498E+1
Difference: Hodges-Lehmann	3.9498E+1

Column B	AAV2.V59
vs.	vs.
Column A	AAV2
Mann Whitney test	
P value	0.0079
Exact or approximate P value?	Exact
P value summary	**
Significantly different (P < 0.05)?	Yes
One- or two-tailed P value?	Two-tailed
Sum of ranks in column A,B	15 , 40
Difference between medians	
Median of column A	24497162657, n=5
Median of column B	253160000000, n=5
Difference: Actual	2.28663E+1
Difference: Hodges-Lehmann	2.28663E+1

Column B	AAV2.V59
vs.	vs.
Column A	AAV-NP59
Mann Whitney test	
P value	0.0952
Exact or approximate P value?	Exact
P value summary	ns
Significantly different (P < 0.05)?	No
One- or two-tailed P value?	Two-tailed
Sum of ranks in column A,B	36 , 19
Difference between medians	
Median of column A	419477000000, n=5
Median of column B	253160000000, n=5
Difference: Actual	-1.66317E+1
Difference: Hodges-Lehmann	-1.66317E+1
